# Antioxidant vitamin index and risk of age-related macular degeneration: multicenter validation and clinical translation

**DOI:** 10.1038/s41514-026-00348-y

**Published:** 2026-02-21

**Authors:** Xuehao Cui, Jingwen Hui, Zheya Han, Quanhong Han

**Affiliations:** 1 Department of Clinical Neurosciences, University of Cambridge, Cambridge, UK; 2 John van Geest Centre for Brain Repair, University of Cambridge, Cambridge, UK; 3 Cambridge Eye Unit, Addenbrooke’s Hospital, Cambridge University Hospitals NHS Foundation Trust, Cambridge, UK; 4 Tianjin Eye Hospital, Tianjin Key Laboratory of Ophthalmology and Visual Science, Tianjin Eye Institute, Tianjin, China; 5 Nankai University Optometry and Vision Sicence Insitute, Nankai University Affiliated Tianjin Eye Hospital, Tianjin, China

**Keywords:** Diseases, Health care, Medical research, Risk factors

## Abstract

Age-related macular degeneration (AMD) is a leading cause of blindness in older adults, with oxidative stress as a central driver. We proposed the Antioxidant Vitamin Index (AVI), a composite indicator integrating vitamins A, C, and E to quantify systemic antioxidant nutritional status, and evaluated its association with AMD across three large cohorts: the UK Biobank, NHANES 2005–2008, and a Tianjin clinical cohort. Using Cox proportional hazards models for incident AMD in the UK Biobank and multivariable logistic regression for AMD prevalence in NHANES and Tianjin, complemented by restricted cubic splines, quartile analyses, and machine learning, we consistently observed that higher AVI was independently associated with a lower risk of AMD. Dose–response analyses showed a progressive decline in AMD risk with increasing AVI, and model performance improved when AVI was added to conventional risk factors. Machine learning and SHAP interpretation further identified age and AVI as dominant predictors of AMD. These findings support AVI as a biologically grounded, quantifiable metric with potential for early screening, risk stratification, and nutrition-based prevention of AMD.

## Introduction

Age-related macular Degeneration (AMD) is a leading cause of vision loss and blindness in older adults. With rapid population aging, the clinical and economic burden of AMD continues to grow and places sustained pressure on health systems^1–4^. The disease progresses gradually, from early drusen and retinal pigmentary changes to intermediate structural damage, and ultimately to late geographic atrophy and macular neovascularization^5–7^. Age is the most substantial risk factor, and smoking, suboptimal dietary patterns, and cardio-metabolic conditions are thought to aggravate the onset and progression^8,9^. Despite advances in imaging and therapy, early identification, quantitative risk stratification, and follow-up planning remain inadequate, underscoring the need for a biologically grounded, quantifiable, and scalable risk metric for population screening and individualized care^10,11^.

Oxidative stress is recognized as a central driver of AMD^12^. The macula resides in an environment of intense light exposure and high oxygen consumption, where photoreceptor outer segments undergo continuous renewal and membranes are rich in oxidizable lipids^13,14^. Photochemical reactions and mitochondrial metabolism continuously generate reactive oxygen species (ROS)^15^. When antioxidant defenses falter, lipid peroxidation, damage to proteins and deoxyribonucleic acid, and dysfunction of the retinal pigment epithelium (RPE) emerge, followed by chronic inflammation, complement dysregulation, remodeling of the Bruch membrane and extracellular matrix, and alterations in the choriocapillaris^16–18^. These processes promote drusen accumulation and fibrotic remodeling^19^. Although geographic atrophy and neovascular phenotypes differ in their late manifestations, both share upstream drivers linked to sustained oxidative and metabolic stress^20,21^.

Enzymatic and nonenzymatic components provide antioxidant protection^22^. The enzymatic system includes superoxide dismutase, catalase, and glutathione peroxidase, which detoxify ROS across cellular compartments^23,24^. The nonenzymatic system relies on diet-derived small molecules in which Vitamin A, Vitamin C, and Vitamin E form a cross-phase network^25,26^. Vitamin A supports the visual cycle and the integrity of photoreceptors and RPE, contributing to transcriptional control and barrier stability, thereby reducing the accumulation of light-induced byproducts at their source^27,28^. Vitamin C acts in aqueous environments to neutralize diverse ROS and to regenerate active Vitamin E, sustaining the antioxidant cycle^29,30^. Vitamin E acts in lipid environments as a chain-breaking antioxidant, protecting membranes enriched with polyunsaturated fatty acids by interrupting lipid peroxidation^31^. Acting together across aqueous and lipid phases, as well as across intra- and extracellular spaces, these vitamins maintain a favorable redox balance under persistent oxidative stress^32^. Complementarity and recycling among them imply that no single vitamin can faithfully represent antioxidant capacity at the individual level, which motivates the use of a composite measure^33,34^.

We therefore introduce the Antioxidant Vitamin Index (AVI), a composite indicator derived from Vitamin A, Vitamin C, and Vitamin E to quantify antioxidant nutritional exposure^35,36^. Several composite indices have been developed to summarize dietary antioxidant exposure, including the Composite Dietary Antioxidant Index (CDAI), the Dietary Antioxidant Quality Score (DAQS), and the Dietary Antioxidant Index (DAI)^37–39^. AVI is intentionally minimalist and retina-focused: it restricts inputs to vitamins A, C, and E, the core aqueous–lipid antioxidant triad supporting the visual cycle and retinal pigment epithelium, assigns them fixed equal weights, and normalizes each intake to its age-appropriate recommended dietary allowance (RDA) before averaging. The scientific rationale of AVI rests on three pillars. First, mechanistic coherence is achieved because AVI maps to the central functional nodes of the antioxidant network, which align with the oxidative stress pathway in AMD^16,40^. Second, statistical robustness is enhanced because a composite measure mitigates random variability in single-nutrient intake and reduces instability arising from differences in total energy intake and dietary patterns, thereby improving reproducibility and transportability across populations^41^. Third, clinical interpretability is enhanced because AVI has a clear biological meaning and can be integrated with age, lifestyle, and routine clinical variables to yield an actionable risk profile. In this study, we validated AVI across three large cohorts from different countries and ancestries, namely the United Kingdom Biobank (UKB), the National Health and Nutrition Examination Survey (NHANES), and a Tianjin cohort. Higher AVI consistently associated with lower AMD risk, indicating its predictive value and potential for clinical translation in early detection, stratified follow-up, and nutrition-guided intervention.

## Results

### Baseline characteristics and correlations

Across the three cohorts, participants with AMD were older than those without the disease, with mean ages of 61.6 years versus 56.0 years in the UKB, 68.6 years versus 56.7 years in the NHANES, and 68.0 years versus 56.4 years in the Tianjin cohort. Males were slightly more frequent among patients, and low income, lower education, and a history of smoking were more common. Diabetes was significantly more prevalent in all datasets, while hypertension was higher in the United Kingdom Biobank (Table 1).

**Table 1:** Baseline characteristics and biochemical, dietary, and nutritional indicators of participants across the UK Biobank, NHANES, and Tianjin cohorts

Cohort	UKB			NHANES			Tianjin		
	HC	AMD	*P*	HC	AMD	*P*	HC	AMD	*P*
*N*	27437	629		2462	351		699	432	
Age	55.97 (7.68)	61.63 (5.99)	**<0.001**	56.74 (11.37)	68.55 (11.66)	**<0.001**	56.43 (10.63)	67.97 (10.58)	**<0.001**
Sex (%)			0.111			0.061			0.03
Male	12592 (45.9)	268 (42.6)		1204 (48.9)	191 (54.4)		326 (46.6)	231 (53.5)	
Female	14845 (54.1)	361 (57.4)		1258 (51.1)	160 (45.6)		373 (53.4)	201 (46.5)	
Race (%)			0.543			**<0.001**			
1	26888 (98.0)	621 (98.7)		1336 (54.3)	245 (69.8)				
2	204 (0.7)	4 (0.6)		466 (18.9)	33 (9.4)				
3	124 (0.5)	1 (0.2)		396 (16.1)	45 (12.8)				
4	221 (0.8)	3 (0.5)		264 (10.7)	28 (8.0)				
Smoke (%)			0.043			**<0.001**			**<0.001**
Never	15964 (58.2)	338 (53.7)		1237 (50.2)	141 (40.2)		360 (51.5)	174 (40.3)	
Former	9752 (35.5)	254 (40.4)		743 (30.2)	148 (42.2)		199 (28.5)	192 (44.4)	
Now	1721 (6.3)	37 (5.9)		482 (19.6)	62 (17.7)		140 (20.0)	66 (15.3)	
Alcohol (%)			0.062			0.472			**0.003**
Never	728 (2.7)	25 (4.0)		272 (22.7)	43 (22.3)		233 (33.3)	187 (43.3)	
Former	713 (2.6)	21 (3.3)		344 (28.7)	48 (24.9)		269 (38.5)	146 (33.8)	
Now	25996 (94.7)	583 (92.7)		582 (48.6)	102 (52.8)		197 (28.2)	99 (22.9)	
Income(PIR)(%)			**<0.001**						
Low	9173 (33.4)	284 (45.2)							
Medium	9785 (35.7)	230 (36.6)							
High	8479 (30.9)	115 (18.3)							
Income(PIR)				2.98 (1.64)	2.59 (1.42)	**<0.001**			
Edu (%)			**<0.001**			**0.001**			
Low	6338 (23.1)	188 (29.9)		863 (35.1)	150 (42.7)				
Medium	7873 (28.7)	176 (28.0)		916 (37.2)	134 (38.2)				
High	13226 (48.2)	265 (42.1)		683 (27.7)	67 (19.1)				
DM (%)			**<0.001**			**0.009**			**0.005**
No	26509 (96.6)	590 (93.8)		2167 (88.0)	291 (82.9)		619 (88.6)	356 (82.4)	
Yes	928 (3.4)	39 (6.2)		295 (12.0)	60 (17.1)		80 (11.4)	76 (17.6)	
HBP (%)			**0.008**			0.648			0.333
No	15778 (57.5)	328 (52.1)		1841 (74.8)	267 (76.1)		523 (74.8)	335 (77.5)	
Yes	11659 (42.5)	301 (47.9)		621 (25.2)	84 (23.9)		176 (25.2)	97 (22.5)	
BMI	26.51 (4.47)	26.68 (4.68)	0.333	28.25 (5.33)	27.85 (4.64)	0.186	28.17 (5.24)	27.96 (4.66)	0.499
Waist	88.31 (13.12)	89.28 (13.01)	0.066	98.23 (13.49)	99.81 (13.18)	**0.039**			
Glu	5.04 (1.13)	5.18 (1.45)	**0.003**				5.75 (1.00)	5.75 (0.85)	0.951
HbA1C	35.27 (5.53)	36.42 (6.03)	**<0.001**	5.75 (1.04)	5.76 (0.85)	0.873			
RBC	4.50 (0.40)	4.50 (0.39)	0.979	4.69 (0.48)	4.64 (0.52)	0.078	4.69 (0.49)	4.64 (0.51)	0.081
WBC	6.60 (1.79)	6.62 (1.77)	0.788				7.12 (1.56)	7.36 (1.74)	**0.017**
PLT	254.68 (57.80)	256.11 (56.33)	0.54				243.02 (52.33)	252.08 (61.19)	**0.008**
MCH	31.44 (1.85)	31.38 (2.05)	0.451						
MCHC	34.60 (1.18)	34.52 (1.47)	0.101						
CRP	2.19 (3.87)	2.68 (5.09)	**0.002**						
TC	5.71 (1.11)	5.71 (1.20)	0.962	5.28 (1.07)	5.21 (1.09)	0.22	5.28 (1.06)	5.31 (1.08)	0.623
TG	1.67 (0.99)	1.62 (0.88)	0.191				1.61 (0.66)	1.65 (0.74)	0.326
ALB	45.32 (2.55)	44.96 (2.54)	**<0.001**	42.17 (2.98)	41.77 (3.04)	**0.02**	42.42 (3.11)	41.69 (2.88)	**<0.001**
ALP	80.53 (23.54)	84.38 (24.41)	**<0.001**	70.79 (21.90)	72.72 (28.53)	0.138	72.90 (22.66)	75.46 (28.70)	0.096
ALT	22.78 (13.31)	22.82 (18.12)	0.929	25.69 (15.22)	23.97 (23.52)	0.067	27.69 (14.41)	27.39 (19.92)	0.772
AST	25.75 (8.84)	26.79 (14.99)	**0.004**	26.25 (13.18)	26.64 (18.35)	0.621	27.73 (11.94)	27.49 (14.90)	0.761
GGT	33.77 (33.69)	36.05 (39.35)	0.095	33.22 (54.17)	31.84 (51.26)	0.652	44.23 (40.68)	40.04 (36.13)	0.079
CREA	71.91 (15.52)	72.12 (25.39)	0.742	81.85 (41.49)	87.57 (26.59)	**0.012**	83.32 (35.04)	90.90 (24.89)	**<0.001**
UREA	5.34 (1.29)	5.64 (1.62)	**<0.001**						
Urate	303.80 (78.39)	305.60 (76.72)	0.568	326.40 (82.93)	342.78 (90.38)	**0.001**	329.31 (82.46)	349.05 (91.73)	**<0.001**
Energy	9059.55 (2533.29)	8735.29 (2396.35)	**0.001**	2008.90 (744.69)	1834.82 (720.05)	**<0.001**	2025.79 (751.40)	1808.60 (717.56)	**<0.001**
Protein	84.03 (25.88)	82.04 (25.14)	0.056	77.90 (33.50)	69.88 (33.23)	**<0.001**	76.76 (32.64)	70.30 (32.08)	**0.001**
Carbohydrate	264.18 (82.86)	255.72 (84.36)	**0.011**	244.58 (102.29)	221.34 (93.48)	**<0.001**	243.71 (105.19)	230.35 (94.43)	**0.031**
Fat	78.81 (30.07)	74.95 (28.16)	**0.001**	75.69 (34.38)	70.51 (34.80)	**0.008**	75.62 (33.74)	71.58 (32.76)	**0.048**
MUFA	29.04 (11.76)	26.17 (10.71)	**<0.001**	27.85 (13.58)	25.88 (13.15)	**0.011**	27.43 (13.63)	26.73 (12.71)	0.389
Vit_A	1100.91 (1255.68)	990.89 (991.60)	**0.029**	642.62 (486.74)	637.79 (510.86)	0.863	693.77 (452.89)	646.87 (448.52)	0.09
Vit_B6	2.16 (0.72)	2.03 (0.66)	**<0.001**	1.93 (1.08)	1.77 (1.01)	**0.007**	1.94 (1.04)	1.75 (0.97)	**0.003**
Vit_B12	6.41 (3.89)	6.31 (3.34)	0.535	5.25 (5.26)	5.04 (5.41)	0.472	5.83 (4.67)	5.84 (4.63)	0.965
Vit_C	134.84 (78.22)	118.71 (78.10)	**<0.001**	82.01 (74.48)	75.68 (66.85)	0.132	91.12 (65.16)	76.25 (57.01)	**<0.001**
Vit_E	11.79 (4.86)	10.42 (4.59)	**<0.001**	7.26 (4.62)	6.73 (4.26)	**0.043**	7.60 (4.49)	6.82 (4.24)	**0.004**
Vit_D	3.82 (3.46)	3.56 (3.09)	0.065						
AVI	1.17 (0.61)	1.04 (0.56)	**<0.001**	0.76 (0.58)	0.67 (0.40)	**0.003**	0.76 (0.29)	0.67 (0.28)	**<0.001**
BVI	2.17 (0.93)	2.10 (0.82)	0.056	1.84 (1.33)	1.73 (1.33)	0.152	1.96 (1.03)	1.89 (1.03)	0.281
CMNI	1.35 (0.55)	1.26 (0.49)	**<0.001**	1.15 (0.64)	1.09 (0.66)	0.094	1.36 (0.53)	1.28 (0.54)	**0.016**
TyG	8.66 (0.56)	8.66 (0.56)	0.981				9.06 (0.50)	9.09 (0.49)	0.346
TyG_BMI	230.43 (47.33)	231.77 (47.67)	0.483						
FIB4	1.30 (0.49)	1.46 (0.50)	**<0.001**						
HSI	34.58 (5.64)	34.59 (5.68)	0.953						
NFS	-2.12 (1.02)	-1.81 (0.98)	**<0.001**						
Fat_Energy_ratio	0.08 (0.02)	0.08 (0.02)	0.13	0.34 (0.09)	0.34 (0.09)	0.107	0.42 (0.42)	0.45 (0.39)	0.275
Protein_Energy_ratio	0.04 (0.01)	0.04 (0.01)	0.118	0.16 (0.05)	0.16 (0.05)	0.223	0.19 (0.17)	0.20 (0.16)	0.528
Carb_Energy_ratio	0.12 (0.02)	0.12 (0.02)	0.996	0.49 (0.11)	0.49 (0.11)	0.512	0.60 (0.52)	0.64 (0.54)	0.246
MUFA_Fat_ratio	0.37 (0.04)	0.35 (0.04)	**<0.001**	0.36 (0.05)	0.37 (0.05)	0.617	0.54 (0.77)	0.64 (1.16)	0.078
LYM				2.12 (0.97)	2.03 (0.73)	0.069			
MONO				0.55 (0.19)	0.59 (0.23)	**<0.001**			
NEU				4.15 (1.61)	4.25 (1.63)	0.244			
HDL_C				1.39 (0.41)	1.42 (0.45)	0.155	1.42 (0.40)	1.45 (0.48)	0.223
Vit_K				106.02 (178.39)	91.47 (147.47)	0.145	132.11 (133.31)	108.24 (114.48)	**0.002**
NLR				2.13 (1.10)	2.36 (1.34)	**<0.001**	2.55 (2.39)	2.76 (2.19)	0.125
Retinol				434.99 (365.02)	437.46 (436.29)	0.908			
Alpha_carotene				423.45 (966.13)	429.07 (821.95)	0.917			
Beta_carotene				2231.08 (3561.23)	2138.75 (3043.57)	0.644			
Beta_crytoxanthin				94.60 (160.61)	98.81 (193.70)	0.655			
HB							144.88 (11.43)	143.49 (13.21)	0.062

Race (%) was harmonised across datasets but originates from distinct coding systems.

In UK Biobank, race categories were defined as 1 = British, 2 = Asian, 3 = Black, and 4 = Other.

In NHANES, race/ethnicity follows the standard NHANES coding: 1 = Non-Hispanic White, 2 = Non-Hispanic Black, 3 = Mexican American, and 4 = Other.

The bolded sections are all P values less than 0.05, which is the standard threshold for statistical significance.

In terms of biochemical indicators, AMD was associated with modest increases in inflammatory and metabolic markers. Levels of CRP, UREA, and ALP were higher in the UKB, while CREA and UA were elevated in the NHANES and Tianjin cohort, indicating mild systemic metabolic stress. Nutritionally, patients with AMD had lower total energy intake and lower consumption of protein, fat, and carbohydrate. Intake of Vitamins A, C, and E, as well as Vitamin B6, was consistently reduced, reflecting a decline in antioxidant nutritional status. The AVI, which combines Vitamins A, C, and E, was significantly lower among patients in all cohorts, with mean values of 1.04 versus 1.17 in the UKB, 0.67 versus 0.76 in the NHANES, and 0.67 versus 0.76 in the Tianjin cohort, all with *P* < 0.01 (Table 1).

Supplementary Fig. S1 demonstrated consistent correlation structures of the AVI across all three cohorts (Supplementary Fig. S1A–C). In the UKB dataset, the AVI showed strong positive correlations with Vitamins A, C, and E, with correlation coefficients ranging between 0.6 and 0.8, forming a distinct antioxidant cluster. A similar pattern was observed in NHANES and the Tianjin cohort. This further confirmed this structure, showing consistent directions of association across different ethnic and dietary backgrounds, indicating excellent reproducibility of the index across populations. In addition, the AVI exhibited weak negative correlations with metabolic and inflammatory markers, including CRP and HbA1C, suggesting that higher antioxidant capacity is associated with lower systemic oxidative and inflammatory burden. The index was also moderately and positively correlated with total energy, protein, and fat intake, highlighting a close link between overall nutritional status and antioxidant capacity.

### Association between the AVI and AMD risk in UKB

**Fig. 1:**
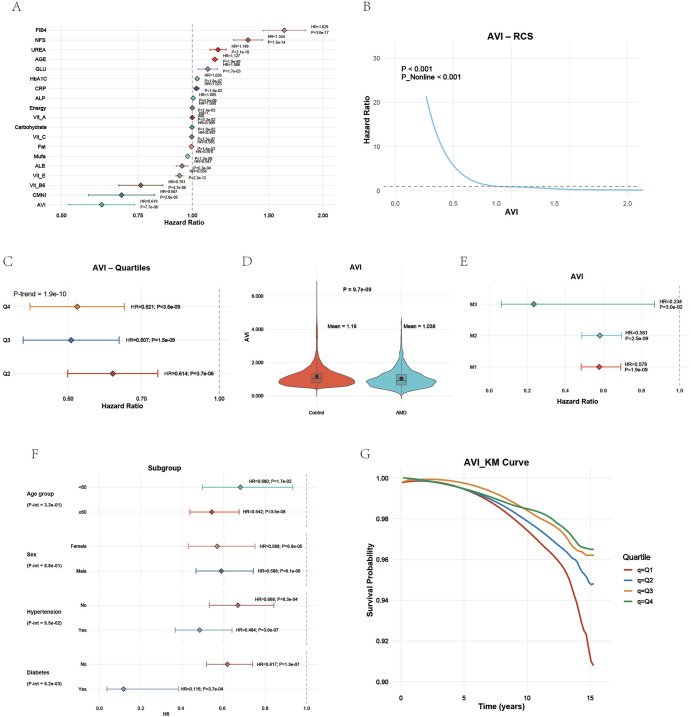
Association between the antioxidant vitamin index and age-related macular degeneration risk in the UK Biobank. **A** Forest plot showing hazard ratios of multiple variables for AMD incidence. AVI demonstrated an independent protective effect. **B** Restricted cubic spline (RCS) curve showing a nonlinear inverse relationship between AVI and AMD risk (*P* < 0.001). **C** Quartile analysis showing stepwise reduction in AMD hazard across increasing AVI groups. **D** Violin plot comparing AVI levels between AMD and control groups. **E** Multivariable models (M1–M3) confirmed AVI’s stable inverse association with AMD. **F** Subgroup analysis showing consistent protective effects across age, sex, hypertension, and diabetes strata. **G** Kaplan–Meier survival curves displaying cumulative AMD-free survival by AVI quartiles.

In the UKB cohort, the AVI showed a strong, independent inverse association with AMD risk. The multivariable Cox regression demonstrated that lower AVI values were linked to a significantly higher hazard of AMD across all adjustment models (Fig. 1A, E). The RCS (Fig. 1B) revealed a pronounced nonlinear dose-response pattern, showing a sharp decline in AMD risk as AVI increased (*P* < 0.001). When divided into quartiles, the risk decreased steadily from Q1 to Q4 (P for trend = 1.9 × 10⁻¹⁰; Fig. 1C). Participants in the highest quartile (Q4) had a 48% lower hazard of AMD compared with Q1 (HR 0.52, *P* = 3.6 × 10⁻⁹), and this association remained significant in the fully adjusted M3 model (HR 0.23, *P* = 3.0 × 10⁻²).

Group comparison indicated that mean AVI values were significantly lower in patients with AMD (1.04) than in controls (1.17), as illustrated in the violin plot (Fig. 1D). Subgroup analyses stratified by age, sex, hypertension, and diabetes showed consistent protective associations across all categories without significant interactions, confirming the robustness of the results (Fig. 1F). The Kaplan–Meier survival curves (Fig. 1G) further supported these findings. Participants in the highest AVI quartile (Q4) exhibited the best AMD-free survival probability, while those in the lowest quartile (Q1) showed the steepest decline over time. Collectively, these results indicate that higher AVI, reflecting stronger antioxidant nutritional capacity, is consistently associated with a lower risk and delayed onset of AMD in the UKB population.

### Validation of the association between AVI and AMD in NHANES

**Fig. 2:**
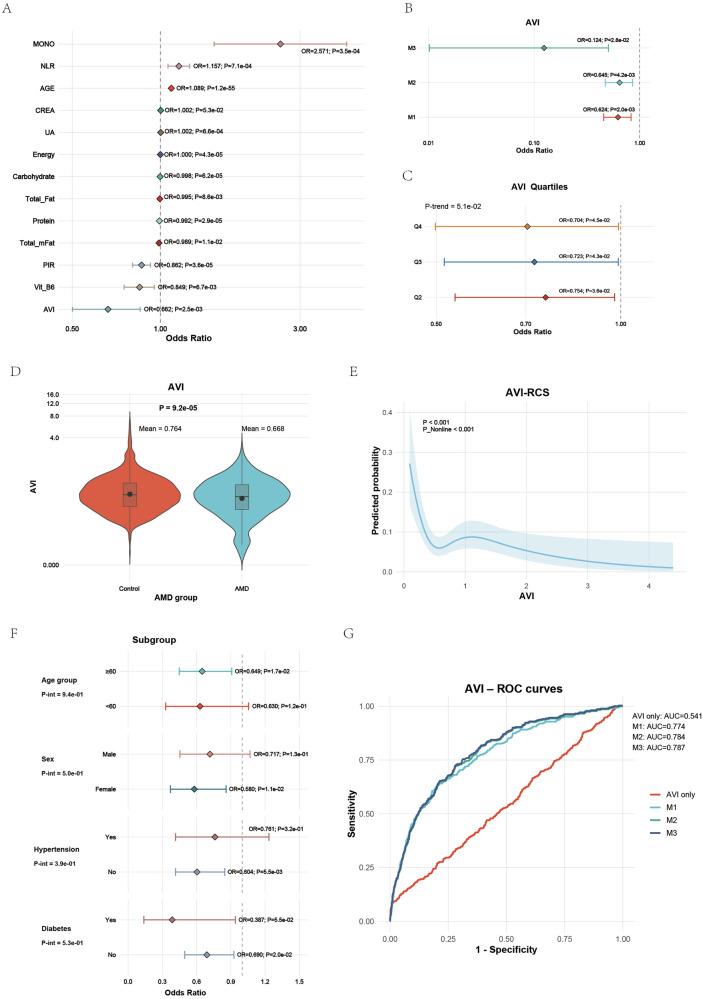
Validation of AVI-AMD association in the NHANES cohort. **A** Forest plot of odds ratios for AMD and associated factors. AVI showed significant negative correlation with AMD risk. **B** Logistic models (M1–M3) indicating consistent protection with higher AVI. **C** Quartile analysis showing decreasing AMD odds across AVI quartiles. **D** Violin plot illustrating lower AVI in AMD participants. **E** RCS analysis showing a nonlinear decline in AMD probability with increasing AVI. **F** Subgroup analyses confirmed robustness across demographics and comorbidities. **G** ROC curves showing progressive improvement in discrimination when AVI was added to predictive models.

In the NHANES cohort, the AVI was independently and negatively associated with AMD. Multivariable logistic regression analyses showed that lower AVI values were linked to higher odds of AMD, and this association remained robust after adjustment for demographic, lifestyle, and metabolic covariates (Fig. 2A, B). When divided into quartiles, the odds of AMD decreased steadily from Q1 to Q4 (Fig. 2C), with participants in Q4 showing a significantly lower risk (OR 0.70, *P* = 4.5 × 10⁻²) and the fully adjusted M3 model confirming a persistent inverse association (OR 0.12, *P* = 2.8 × 10⁻²). The mean AVI level was markedly lower in AMD cases (0.67) compared with controls (0.76), indicating reduced antioxidant capacity (Fig. 2D). The restricted cubic spline (Fig. 2E) further demonstrated an apparent nonlinear decline in AMD probability with increasing AVI (*P* < 0.001).

Subgroup analyses stratified by age, sex, hypertension, and diabetes yielded consistent results, showing similar protective directions of AVI across all categories without significant interactions (Fig. 2F). Model discrimination analysis showed good predictive performance, with the area under the receiver operating characteristic curve (AUC) improving from 0.541 for AVI alone to 0.774, 0.784, and 0.787 for Models 1–3, respectively (Fig. 2G). These results confirm that higher AVI, reflecting stronger antioxidant nutritional status, is consistently associated with a lower likelihood of AMD in the NHANES population, supporting the reproducibility of findings across diverse populations.

### Validation of the association between AVI and AMD in the Tianjin cohort

**Fig. 3:**
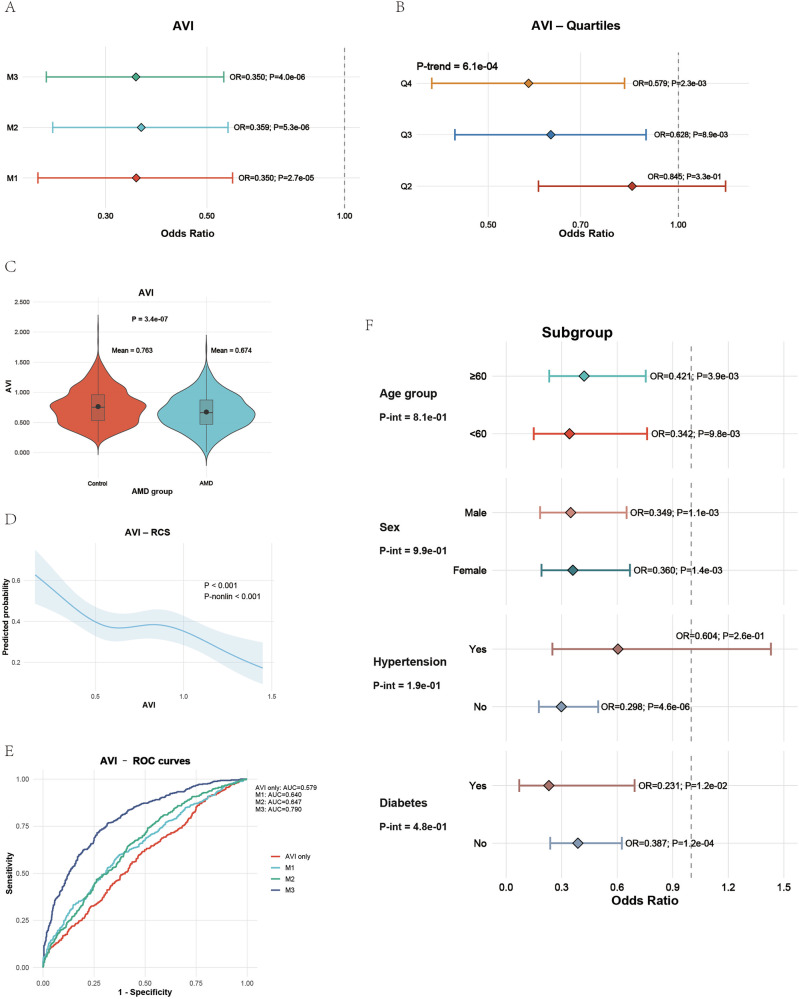
External validation of AVI in the Tianjin cohort. **A** Multivariable logistic regression showing an inverse association between AVI and AMD risk across all models. **B** Quartile analysis with significant linear trend (*P* for trend = 6.1 × 10^−4^). **C** Violin plot indicating lower AVI among AMD cases. **D** RCS analysis revealing a negative nonlinear relationship between AVI and AMD probability. **E** ROC analysis demonstrating increasing predictive accuracy with model adjustment. **F** Subgroup analyses showing consistent protection, with stronger effects in participants without hypertension or diabetes.

In the Tianjin cohort, the AVI showed a consistent and significant inverse relationship with AMD. Multivariable logistic regression analyses demonstrated that lower AVI values were associated with higher odds of AMD, and this association remained robust after adjusting for demographic, metabolic, and clinical covariates (Fig. 3A). When divided into quartiles, the odds of AMD decreased progressively from Q1 to Q4 (*P* for trend = 6.1 × 10⁻⁴; Fig. 3B), with participants in Q4 showing significantly lower odds (OR 0.58, *P* = 2.3 × 10⁻³) than those in Q1. This inverse relationship persisted in the fully adjusted M3 model (OR 0.35, *P* = 4.0 × 10⁻⁶). The mean AVI was lower in AMD cases (0.67) than in controls (0.76), confirming reduced antioxidant capacity (Fig. 3C). The restricted cubic spline (Fig. 3D) further illustrated a nonlinear decline in AMD probability with increasing AVI (*P* < 0.001).

The model’s predictive performance was satisfactory, with the area under the receiver operating characteristic curve (AUC) increasing from 0.579 for AVI alone to 0.680, 0.744, and 0.760 for the progressively adjusted models (Fig. 3E). Subgroup analyses by age, sex, hypertension, and diabetes confirmed consistent protective effects of AVI across all categories, with stronger associations observed in participants without hypertension or diabetes and no significant interactions detected (Fig. 3F). Overall, these findings validate the reproducibility of the AVI-AMD relationship in an independent Chinese cohort. Higher AVI, reflecting stronger antioxidant nutritional status, was associated with a significantly reduced risk of AMD, reinforcing the biological plausibility and global applicability of the index across different populations.

To further determine whether the predictive advantage of the AVI could be reproduced by its individual components, we conducted additional ROC analyses in the NHANES and Tianjin cohorts by modeling vitamin A, vitamin C, and vitamin E separately. Specifically, each vitamin was entered individually as the sole antioxidant exposure within the fully adjusted Model 3 framework, without inclusion of the AVI or the other two vitamins. As shown in Supplementary Fig. S8, single-vitamin models demonstrated limited discriminative ability in both cohorts, with AUC values close to 0.50–0.54. Even under full multivariable adjustment, Model 3 incorporating only one antioxidant vitamin yielded substantially lower AUCs (~0.69–0.71) compared with the corresponding Model 3 that included the AVI (AUC ≈ 0.79 in NHANES and ≈0.79 in the Tianjin cohort; Figs. 2G and 3E). These findings indicate that no individual vitamin alone can recapitulate the predictive performance of the AVI, and that the composite index captures synergistic antioxidant information beyond what is conveyed by any single component.

### Feature selection and risk prediction model in UKB

**Fig. 4:**
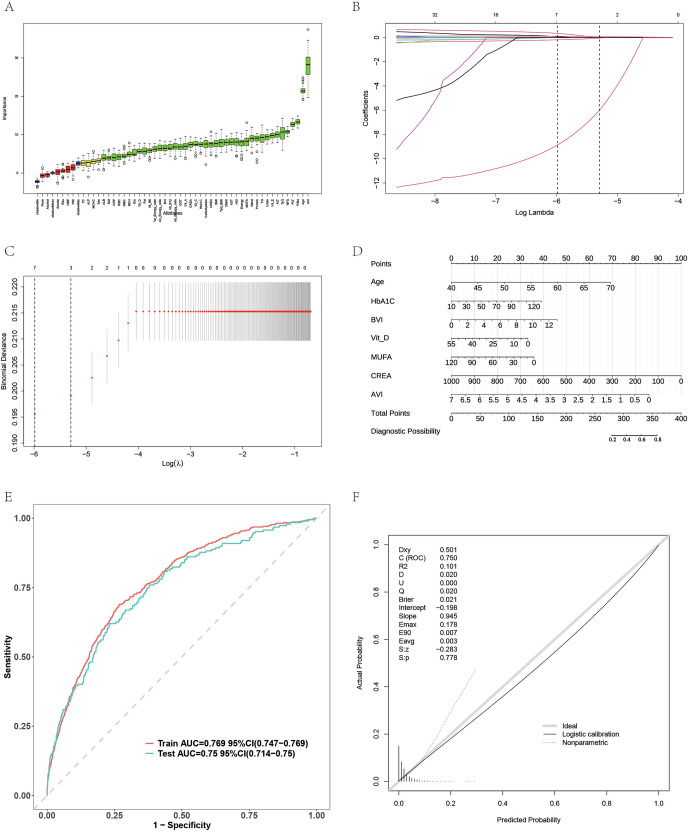
Feature selection and AMD risk prediction in UKB. **A** Boruta algorithm ranking feature importance. **B**, **C** LASSO coefficient paths and cross-validation showing key predictors retained. **D** Nomogram integrating age, HbA1c, BVI, Vitamin D, MUFA ratio, creatinine, and AVI for individual AMD risk estimation. **E** ROC curves showing high discriminative power (AUCtrain=0.769, AUCtest = 0.750). **F** Calibration curve demonstrating strong alignment between predicted and observed outcomes.

In the UKB cohort, both the Boruta and LASSO methods identified age and AVI as the most important predictors of AMD. The Boruta algorithm (Fig. 4A) ranked AVI and age as the top contributors, and the LASSO regression (Fig. 4B, C) confirmed their stability under penalized model selection. A nomogram integrating key variables, including age, HbA1c, B-vitamin index, vitamin D, MUFA ratio, creatinine, and AVI, was developed to predict AMD risk at the individual level (Fig. 4D). The model exhibited good discriminative performance with an area under the ROC curve (AUC) of 0.769 in the training set and 0.750 in the test set (Fig. 4E). The calibration plot demonstrated close alignment between predicted and observed probabilities, indicating excellent model reliability (Fig. 4F). These results highlight that AVI, when combined with clinical and nutritional indicators, enhances the accuracy of AMD risk prediction in the UKB population.

### Feature selection and risk prediction model in NHANES

**Fig. 5:**
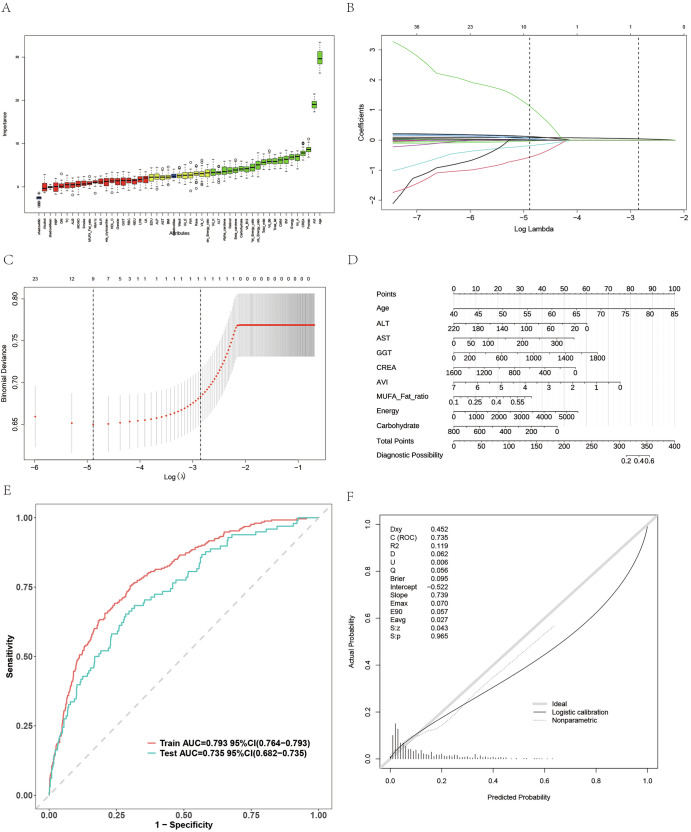
Feature selection and prediction model performance in NHANES. **A** Boruta and **B**, **C** LASSO analyses identified age and AVI as key features. **D** Nomogram combining metabolic, nutritional, and biochemical variables for AMD risk estimation. **E** ROC curves showing strong discrimination (AUCtrain = 0.793, AUCtest = 0.735). **F** Calibration plot confirming good model reliability.

In the NHANES cohort, both Boruta and LASSO analyses identified age and AVI as the most influential predictors of AMD. The Boruta results (Fig. 5A) highlighted the dominant importance of AVI and age, while LASSO regression (Fig. 5B, C) confirmed their contribution to model stability. The nomogram constructed using age, ALT, AST, GGT, creatinine, AVI, MUFA ratio, energy, and carbohydrate intake (Fig. 5D) demonstrated a reliable predictive capacity. Model performance showed AUC values of 0.793 in the training set and 0.735 in the test set (Fig. 5E), with good calibration agreement (Fig. 5F). These findings confirm that AVI, together with metabolic and nutritional variables, provides an effective and interpretable prediction of AMD in the NHANES population.

### Feature selection and risk prediction model in Tianjin cohort

**Fig. 6:**
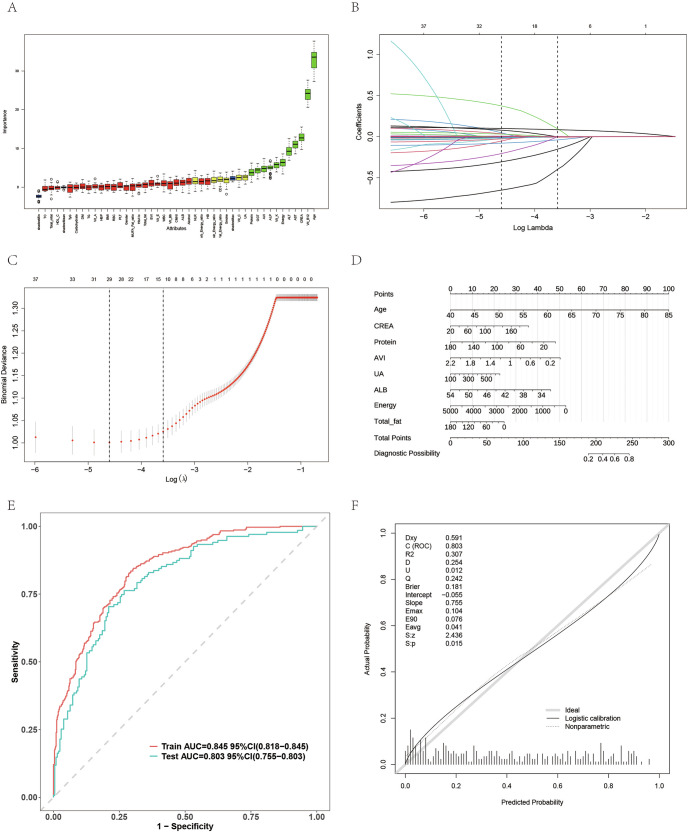
Feature selection and risk prediction model in the Tianjin cohort. **A**–**C** Feature importance identified by Boruta and LASSO showing age and AVI as leading predictors. **D** Nomogram integrating age, creatinine, protein intake, AVI, uric acid, albumin, energy, and total fat for individualized AMD risk assessment. **E** ROC curves showing high discriminative performance (AUCtrain = 0.845, AUCtest = 0.803). **F** Calibration plot demonstrating accurate predictive performance.

In the Tianjin cohort, Boruta and LASSO analyses again highlighted age and AVI as key predictors of AMD. The Boruta ranking (Fig. 6A) placed age at the top, followed by AVI, and the LASSO coefficient path (Fig. 6B, C) confirmed their consistency. The nomogram, which integrates age, creatinine, protein intake, AVI, uric acid, albumin, energy, and total fat (Fig. 6D), provides individualized AMD risk estimates. The model achieved excellent performance with AUC values of 0.845 in the training set and 0.803 in the test set (Fig. 6E), and good calibration consistency (Fig. 6F). These results demonstrate that AVI, in conjunction with nutritional and metabolic indicators, can effectively predict AMD risk in the Chinese population, further validating its clinical applicability.

### Machine learning-based prediction and feature interpretation across cohorts

Machine learning models were applied to the UKB, NHANES, and Tianjin datasets to evaluate the predictive value of the AVI for AMD comprehensively. Four algorithms were compared, including logistic regression, support vector machine (SVM), random forest, and extreme gradient boosting (XGBoost). A compact overview of the algorithms, key hyperparameters, data split, and test performance for each cohort is provided in Supplementary Table S3. Across all three cohorts, tree‑based ensemble methods (random forest and XGBoost) and SVM showed comparable or superior discrimination to logistic regression, with XGBoost achieving the highest or near‑highest AUC in UKB and NHANES, and competitive performance in the Tianjin cohort.

**Fig. 7:**
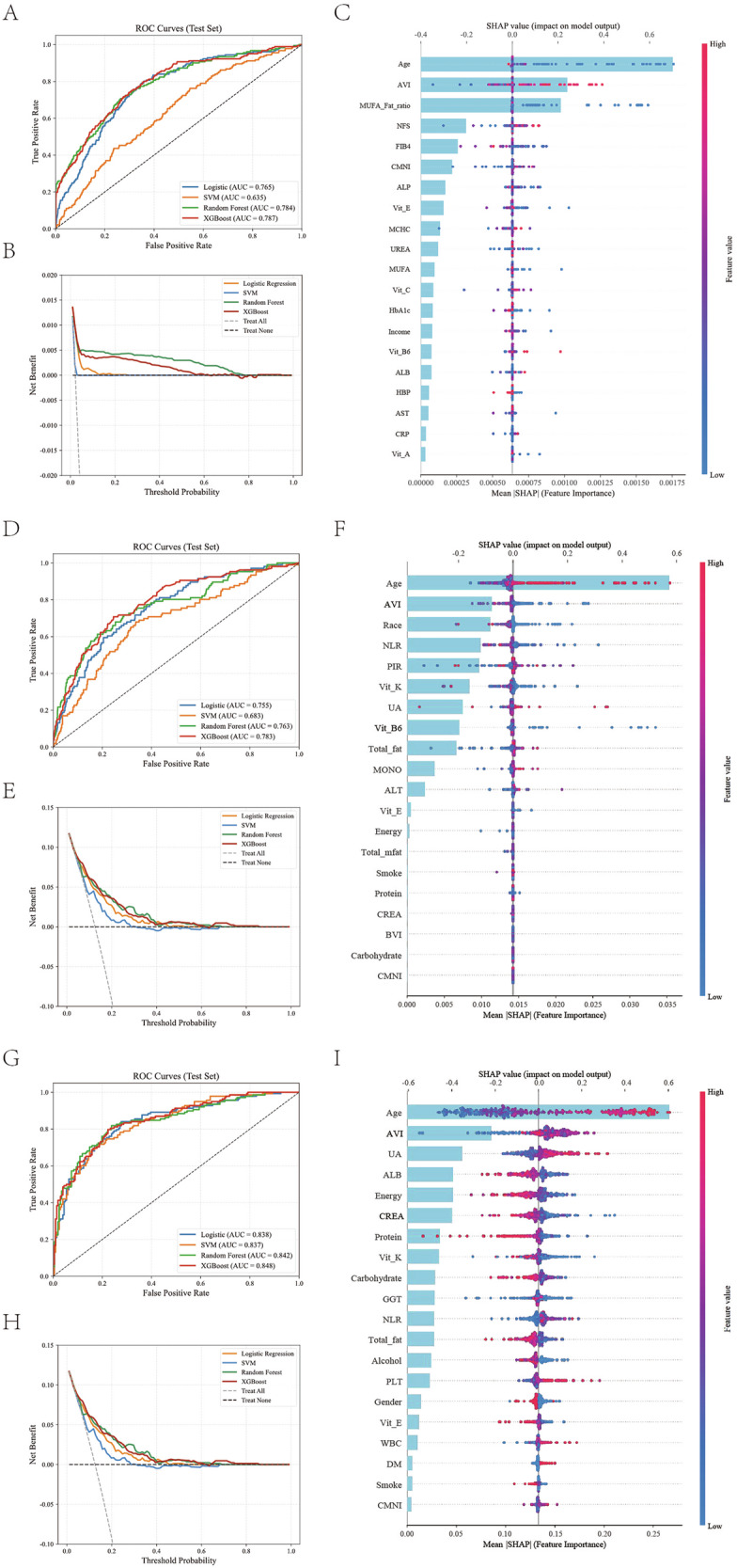
Machine-learning-based risk prediction and model interpretation across cohorts. **A**–**C** correspond to the UK Biobank, **D**–**F** to NHANES, and **G**–**I** to the Tianjin cohort. Each row represents one cohort, displaying the ROC curves, decision-curve analyses, and SHAP feature-importance plots, respectively.

In the UKB dataset (Fig. 7A–C), XGBoost achieved the highest discrimination ability with an area under the ROC curve (AUC) of 0.787, followed by random forest (AUC 0.784), logistic regression (AUC 0.765), and SVM (AUC 0.635). Decision curve analysis showed that XGBoost provided the most significant net clinical benefit (Fig. 7B). SHAP interpretation plots revealed that age and AVI were the most influential variables, followed by MUFA ratio, NFS, and FIB4 (Fig. 7C). In NHANES (Fig. 7D–F), XGBoost again showed superior performance (AUC 0.783) compared with random forest (AUC 0.763), logistic regression (AUC 0.755), and SVM (AUC 0.683). SHAP plots confirmed that age and AVI remained the two most important predictors, while race, NLR, and income contributed to model variation (Fig. 7F). In the Tianjin cohort (Fig. 7G–I), XGBoost achieved the best AUC (0.848), comparable to SVM (0.857) and random forest (0.842), and outperformed logistic regression (0.833). Decision curve analysis (Fig. 7H) indicated that inclusion of AVI significantly improved the clinical utility of the prediction model. SHAP values showed that age and AVI contributed most to the feature contribution spectrum, followed by uric acid, albumin, and total energy (Fig. 7I). Supplementary Figs. S2–S7 further visualized variable interactions and feature importance across cohorts. Pairwise SHAP dependence plots highlighted synergistic effects between AVI and other nutritional or metabolic factors, such as Vitamin E, MUFA ratio, and energy intake, as well as modest interactions between inflammatory markers and oxidative stress–related variables. These findings consistently confirm that AVI, a marker of systemic antioxidant status, is among the most stable and biologically interpretable predictors of AMD across diverse populations and analytical frameworks.

## Discussion

In this study, we introduced AVI, a novel composite indicator combining vitamins A, C, and E. We demonstrated a significant inverse association between AMD risk and this factor across three large, diverse cohorts. Higher AVI scores were consistently associated with lower AMD incidence or prevalence in the UK Biobank, NHANES, and a Chinese Tianjin clinical cohort, even after adjusting for known risk factors. Notably, this inverse relationship was observed using multiple analytical approaches, including Cox proportional hazards models for longitudinal data, logistic regression for cross-sectional comparisons, and machine learning classification, all of which confirmed that AVI independently predicts a reduced risk of AMD. These convergent findings across methodologies and populations underscore the robustness and generalizability of the association between higher antioxidant vitamin status and a lower risk of AMD.

Methodologically, AVI builds on, but also departs from, existing composite antioxidant indices widely used in nutritional epidemiology. CDAI, DAI, and DAQS were designed to reflect overall dietary antioxidant potential or systemic oxidative balance and typically integrate 5–7 antioxidant nutrients. These indices sum equally weighted nutrient-specific z-scores or assign 0/1 points based on whether intakes exceed a fraction of the recommended daily intake, generating population-standardized scores that are well-suited for global redox profiling but more challenging to interpret mechanistically at the organ level and not directly comparable between cohorts^42^. Unlike these composite antioxidant indices, AVI uses only vitamins A, C, and E normalized to RDAs. This yields a mechanistically coherent and biologically interpretable measure that directly maps to the cross-phase aqueous–lipid antioxidant network and the visual cycle in the retina. Conceptually, our work borrows from CDAI/DAQS/DAI the idea of summarizing multiple antioxidant nutrients into a single continuous score and, similar to DAQS, anchors the metric in recommended intakes. In contrast, AVI is intentionally minimalist and retina-focused: it restricts inputs to vitamins A, C, and E, the core aqueous–lipid antioxidant triad supporting the visual cycle and retinal pigment epithelium, assigns them fixed equal weights, and normalizes each intake to its age-appropriate RDA before averaging. This design yields a mechanistically coherent and biologically interpretable measure that maps directly onto the aqueous–lipid antioxidant cycle and retinal oxidative pathways, while providing a continuous dimensionless score in which AVI = 1.0 indicates that, on average, RDA-level intake of the three vitamins is achieved. Values are directly comparable across cohorts and measurement platforms.

The macula operates under intense light and high oxygen demand^43^. At the same time, photoreceptor outer segments are enriched with polyunsaturated lipids, and the RPE undertakes daily phagocytosis and the visual cycle^44,45^. This configuration makes oxidative stress a principal driver of AMD. Persistent ROS initiates membrane lipid peroxidation, mitochondrial dysfunction, complement activation, and the accumulation of phototoxic bisretinoids and lipofuscin, which amplify inflammation and metabolic imbalance in RPE^46^. The AVI integrates Vitamins A, C, and E into a single cross-phase metric that maps directly onto this biology. Vitamin E resides in lipid domains as a chain-breaking antioxidant that terminates peroxyl radicals and protects membranes of photoreceptor discs and the apical microvilli of the RPE^47^. Vitamin C acts in aqueous compartments to neutralize diverse oxidants and regenerate oxidized vitamin E to its active form through the ascorbate-tocopheroxyl redox couple, thereby sustaining the efficiency of lipid-phase defense while supporting small-molecule redox systems^48^. Vitamin A sustains the visual cycle and limits overflow of all-trans retinal toward lipofuscin precursors, and retinoic acid modulates transcriptional programs that maintain epithelial barrier integrity and antioxidant enzyme expression^49^. These vitamins occupy compartments and layers of control, forming a continuous and mutually reinforcing network^50^. A deficit at any node weakens the entire cycle, which explains why single vitamins insufficient proxies for ocular redox resilience are and why a composite index is a better representation of actual physiology. The nonlinear relationship between the AVI and disease risk offers further mechanistic insight. The steep decline in risk at low index values suggests that small increases in antioxidant pools can markedly suppress membrane peroxidation and visual cycle byproducts when the system is depleted. In contrast, marginal benefits diminish as aqueous and lipid pools approach saturation. Model interpretation showed cooperative patterns between the AVI and nutritional factors such as the monounsaturated fat ratio, energy, and protein, consistent with the influence of membrane unsaturation and whole-body metabolism on oxidative and inflammatory tone^51^. Systemic-to-ocular transport provides a biological bridge, allowing systemic exposure to serve as a proxy for retinal availability: retinol-binding protein delivers Vitamin A, lipoproteins carry Vitamin E, and sodium-dependent transporters move Vitamin C into retinal cells^52^. Together, these lines of evidence support the AVI as a quantitative surrogate of ocular antioxidant capacity and a mechanistic link to the pathogenesis of AMD.

Our results address and build upon prior mixed findings in the literature regarding the individual effects of nutrients on AMD. Many earlier studies have examined vitamins A, C, or E in isolation, often yielding inconsistent or modest associations with AMD risk^53–56^. For instance, some observational analyses have reported that high dietary intake or serum levels of vitamin E or vitamin C alone are not significantly associated with lower AMD incidence, potentially due to confounding factors or an insufficient effect size when considered in isolation^57^. Similarly, vitamin A alone has shown variable results across studies, and concerns about beta-carotene’s risk in smokers have complicated its standalone use. These inconsistencies highlighted the limitations of single-nutrient approaches in a disease as multifactorial as AMD. Our introduction of AVI as a composite index overcomes some of these limitations by accounting for the combined intake/level of three major antioxidant vitamins simultaneously. This approach is supported by the concept that nutrients in a typical diet interact and may have synergistic effects - we consume vitamins together in foods, not in isolation. By aggregating the antioxidant contributions of Vit_A, Vit_C, and Vit_E, AVI provides a more integrative nutritional exposure metric that improves the signal-to-noise ratio for detecting diet-disease relationships. In fact, our analysis revealed that the individual association of each vitamin with AMD was weak when adjusted for the others, but the composite AVI showed a clear protective relationship. This finding aligns with emerging nutritional epidemiology frameworks that emphasize dietary patterns or composite scores rather than individual nutrients as better at capturing the actual impact of nutrition on chronic diseases. Thus, our study extends prior research by demonstrating that a combined AVI can reveal a strong inverse association with AMD risk that single-vitamin analyses might miss.

To our knowledge, this study is the first to propose AVI as an aggregated measure of antioxidant vitamin status and to validate its relevance to AMD across multiple populations. The originality of our work lies not only in formulating this index but also in corroborating its association with AMD in diverse cohorts spanning different ethnicities and geographic regions. We confirmed the protective association of high AVI in a large European-descendant population, in a representative United States population sample, and in an East Asian population. The consistency of results across these cohorts is remarkable, considering the differences in dietary habits, genetic backgrounds, and AMD assessment methods among them^58,59^. This broad validation enhances the credibility and generalizability of AVI as a risk indicator. It suggests that the relationship between antioxidant vitamin levels and AMD is fundamental and transcends specific lifestyles or ethnic contexts. Moreover, by successfully applying AVI across disparate datasets, our study demonstrates the feasibility of a unified nutritional index for international research. This cross-population approach is a significant strength, as previous nutrition-AMD studies have often been confined to single cohorts or Western populations. Our multi-ethnic findings contribute novel evidence that antioxidant nutrition is universally relevant to AMD risk, and they highlight the innovation of using a composite vitamin index as an epidemiological tool. As an observational study, our findings should not be interpreted as proof of causality. Future research, including MR and randomized nutrition interventions, is needed to triangulate causal evidence and determine whether increasing AVI can directly reduce AMD risk.

AVI is a straightforward and actionable indicator of ocular risk that can be derived from dietary or serum data. It supports early identification of high-risk individuals, enables stratified follow-up and nutrition-based interventions, and can be integrated into electronic records and community programs. AVI provides a practical target for enhancing dietary quality in older adults and promoting nutrition-centered secondary prevention. Priorities include randomized or pragmatic trials to test whether raising AVI reduces incidence or slows progression, as well as multi-omics and genetic epidemiology to strengthen causality and identify susceptible subgroups. Additionally, there is a focus on developing actionable thresholds and clinical pathways, evaluating expanded indices that include carotenoids and zinc, and considering cost-effectiveness.

Beyond its epidemiological association, the AVI may have important implications for precision medicine in AMD. Oxidative stress is not uniformly distributed across AMD phenotypes; instead, it contributes differentially to the pathogenesis of early disease, geographic atrophy, and macular neovascularization. By quantifying systemic antioxidant vitamin capacity in a standardized manner, AVI offers a biologically interpretable axis for patient stratification. Individuals with low AVI may represent a subgroup with heightened oxidative vulnerability, who could derive greater benefit from targeted nutritional optimization or closer surveillance. Moreover, AVI integrates seamlessly with multi-modal risk frameworks that incorporate age, genetic susceptibility, metabolic status, and imaging-derived biomarkers. Its consistent importance across machine learning models and cohorts suggests that it captures a stable, cross-population signal rather than a context-specific dietary effect. In future studies, AVI could be combined with genotype-informed risk scores, retinal imaging features, or inflammatory markers to delineate biologically meaningful AMD subtypes and to guide personalized prevention strategies. Such an approach aligns with emerging paradigms in ophthalmic precision medicine, shifting from single-nutrient or single-pathway models toward integrative, mechanism-driven risk stratification.

This study benefits from a large sample and a comprehensive design. By combining three primary cohorts and analyzing tens of thousands of participants, we achieved strong statistical power, allowing us to examine the relationship between AVI and AMD across diverse contexts. We applied multiple analytical approaches, including Cox models for incidence, logistic models for prevalence, and machine learning for pattern recognition. The convergent results from these approaches strengthened our inference and robustness. We also adjusted rigorously for confounders such as age, sex, smoking status, BMI, and other health and lifestyle factors, showing that the association between higher AVI and lower AMD risk is independent of these variables. The multiethnic design enhances generalizability, as consistent results across European, North American, and Asian populations suggest a broadly relevant biological role of antioxidant vitamins in AMD prevention.

Several limitations should be acknowledged. The observational design precludes causal inference, and individuals with higher AVI may differ in other health-related behaviors or baseline characteristics that are not fully captured despite extensive adjustment. Measurement heterogeneity and residual confounding are also unavoidable across cohorts. In the UK Biobank and NHANES, AVI was derived exclusively from self-reported dietary intakes of vitamins A, C, and E, which are subject to recall bias, systematic under-reporting, and within-person variability, whereas the Tianjin cohort incorporated clinically assessed dietary information that also reflected supplement use. Although intake data were harmonized to daily amounts, energy-adjusted, and scaled using a unified RDA-based algorithm, differences in dietary assessment instruments, recall windows, and food-composition databases may introduce heterogeneity. Under non-differential misclassification, such errors would be expected to attenuate true associations, suggesting that the observed effects may be conservative; however, in the clinic-based Tianjin cohort, post-diagnosis changes in diet or supplement use and differential recall cannot be entirely excluded. In addition, although some cohorts include blood measurements of vitamins A, C, and E, we were unable to construct and formally compare a serum-based AVI, and thus cannot determine whether a biomarker-defined index would perform better than diet-derived measures. AVI focuses on three antioxidant vitamins and does not capture other nutrients relevant to macular health, such as lutein, zeaxanthin, zinc, or long-chain polyunsaturated fatty acids, and may therefore partially proxy broader dietary patterns; these components were not included due to limited harmonized data and only partial mechanistic overlap with the vitamin A/C/E network. AMD ascertainment also differed across datasets, ranging from record-based diagnoses to imaging-based clinical assessment, and disease stage was not uniformly classified into early and late forms. Finally, the hospital-based Tianjin cohort may not fully represent the general population, and vitamin supplementation data varied substantially across cohorts: Tianjin AVI reflects combined dietary and supplemental intake, whereas in the UK Biobank and NHANES supplementation was recorded mainly as binary use, precluding precise quantification and construction of a fully supplementation-adjusted AVI. Future large-scale prospective studies and randomized trials with standardized, quantitative assessment of diet, supplements, and circulating biomarkers will be required to further refine and validate antioxidant indices.

In conclusion, across three diverse cohorts, AVI shows a consistent and independent inverse association with AMD. It captures the synergy of antioxidant defenses, delivering predictive and translational value. As a quantifiable, nutrition-based metric, AVI offers a new tool for early screening, risk stratification, and personalized intervention in retinal degenerative diseases.

## Methods

### Study design and data sources

**Fig. 8:**
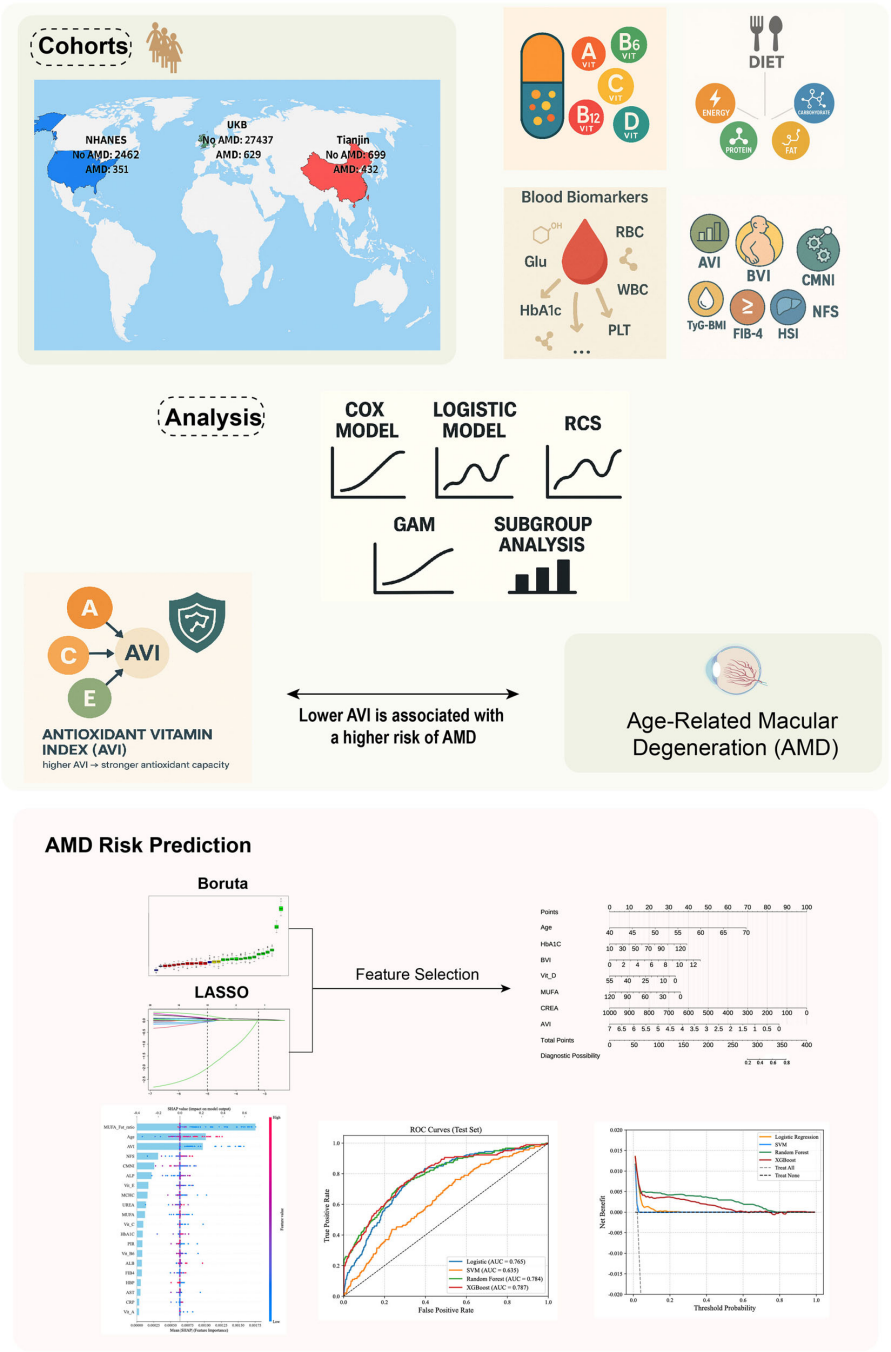
Overview of study design and analytical framework. This schematic illustrates the multicenter study design integrating three major cohorts-UK Biobank (UKB), NHANES (2005–2008), and the Tianjin clinical cohort. Key variables included dietary intake, blood biomarkers, and composite nutritional indices such as the Antioxidant Vitamin Index (AVI), B-vitamin index (BVI), and Comprehensive Macronutrient Index (CMNI). Analytical methods included Cox regression, logistic regression, restricted cubic spline (RCS), generalized additive modeling (GAM), and subgroup analyses. AVI was constructed from Vitamins A, C, and E to quantify antioxidant capacity. Feature selection with Boruta and LASSO, model building, and machine learning algorithms (Logistic, SVM, Random Forest, XGBoost) were used for AMD risk prediction and clinical evaluation.

We implemented a multicenter, multi-cohort framework with discovery, validation, and extension stages (Fig. 8). Three independent datasets were analyzed to ensure robustness and transportability. The UKB prospective cohort was used to examine incident AMD as a time-to-event outcome. NHANES 2005–2008 provided cross-sectional validation of dietary and nutrient exposures in relation to AMD status. The Tianjin cohort included clinic-based AMD cases diagnosed at Tianjin Eye Hospital between January 2020 and January 2025 and controls with non-retinal conditions, such as cataract. In Tianjin, detailed information on dietary intake and vitamin use was collected through structured questionnaires during clinical visits (Supplementary Tables S1, S2). All datasets included demographics, lifestyle factors, laboratory biomarkers, and harmonized dietary and vitamin data, enabling the construction of composite exposure indices. Written informed consent was obtained at enrollment for the Tianjin cohort, with local ethics approval granted. The overall workflow is summarized in Fig. 8.

AMD ascertainment differed across cohorts but was harmonized for comparability. In UKB, incident AMD was identified from linked hospital admission and mortality records using ICD-10 code H35.3, with follow-up from baseline (2006–2010) to 2023 or death; participants with baseline AMD were excluded. Because ICD-10 H35.3 does not differentiate early from late AMD, all stages were analyzed together, and sensitivity analyses excluding suspected late AMD yielded consistent findings. In NHANES, AMD status was defined by fundus photography grading using AREDS criteria and by self-reported diagnosis. In the Tianjin cohort, AMD was clinically confirmed by ophthalmologists using OCT and fundus examination. Early and late AMD were combined to maximize statistical power, with sensitivity analyses supporting consistent directions across subtypes.

Additional details on retinal imaging and AMD grading are as follows. In UKB, spectral-domain OCT and non-mydriatic 45° color fundus photographs were obtained in a subset of participants using the Topcon 3D OCT-1000 Mark II system; however, these images were not used for AMD definition, which relied solely on ICD-10 H35.3 records. In NHANES 2005–2008, participants aged ≥40 years underwent bilateral 45° non-mydriatic digital fundus photography with a Canon CR6-45NM camera. Images were graded at a central reading center using a standardized protocol based on the modified Wisconsin Age-Related Maculopathy Grading System and AREDS definitions, with masked graders and adjudication of discrepancies by a senior grader; participant-level AMD status was assigned based on the worse eye. In the Tianjin cohort, all participants received dilated fundus examination, spectral-domain OCT (Spectralis OCT, Heidelberg Engineering), and color fundus photography (Topcon). AMD was graded according to AREDS-based criteria by two masked ophthalmologists, with disagreements resolved by a third senior retinal specialist.

### Exposure and outcome definitions

The primary exposure was AVI. AVI integrates Vitamin A, Vitamin C, and Vitamin E after energy adjustment and standardization using a prespecified algorithm to quantify composite antioxidant nutritional exposure. Detailed specifications for normalization, weighting, and calculation are provided in the Supplement. To ensure consistency across cohorts, the AVI calculation was standardized and is now fully reported here. AVI = mean [(Vitamin A/900 μg RAE), (Vitamin C/90 mg), (Vitamin E/15 mg)], where 900 μg, 90 mg, and 15 mg represent the recommended dietary allowances (RDAs) for adults. Vitamin intakes in both the UK Biobank and NHANES were obtained from 24-hour dietary recalls and food frequency questionnaires, and were expressed as daily intakes after energy adjustment using the residual method. The Tianjin cohort adopted the same approach as NHANES, collecting dietary intake information through structured dietary recalls and food-frequency questionnaires (Supplementary Tables S1, S2). This harmonized design ensured comparability across datasets. Higher AVI values indicate stronger overall antioxidant vitamin status. We evaluated weighting schemes based on antioxidant potency and bioavailability, but unweighted averaging showed higher cross-cohort reproducibility and was therefore used.

Comparator and extension exposures included the B vitamin index (BVI), a comprehensive macronutrient index (CMNI), the triglyceride glucose index (TyG), body mass index (BMI) adjusted TyG (TyG_BMI), liver fibrosis and steatosis related scores (FIB4, HSI, NFS), energy composition ratios for fat, protein and carbohydrate, and the ratio of monounsaturated fat to total fat (MUFA_Fat_ratio). These indices were computed using established equations or approaches analogous to AVI, as detailed in the Supplement.

Dietary and vitamin assessments were obtained from cohort-specific questionnaires or recalls in the UKB and the Tianjin cohort, as well as from twenty-four-hour dietary recalls and laboratory assays in NHANES. Intakes were harmonized to daily units across datasets and adjusted for total energy. Energy was included as a covariate in primary models. Nutrient density and the residual method were examined in sensitivity analyses.

The primary outcome in UKB was time to first AMD diagnosis ascertained from linked records, with follow-up from baseline to incident AMD, death, or end of follow-up. In NHANES and the Tianjin cohort, the outcome was AMD status determined by retinal imaging and clinical diagnosis. For cross-cohort comparability, AMD was analyzed as a binary endpoint.

### Covariates, eligibility, and data preprocessing

Covariates were organized into a three-level adjustment scheme. Model 1 included age, sex, and race or ancestry composition. Model 2 added education, BMI, waist circumference, smoking, alcohol consumption, diabetes, and hypertension. Model 3 was the complete model that further incorporated laboratory biomarkers, total energy intake, and other clinical variables relevant to AMD. Eligibility criteria excluded participants with missing AMD status, missing primary exposures or key covariates, or implausibly high or low energy intake. For prospective analyses in UKB, individuals with AMD at baseline were excluded. Data harmonization aligned units for continuous variables across cohorts. Right-skewed variables were log or Box–Cox transformed. Extreme values were handled by percentile trimming or winsorization. Categorical variables were consistently recorded. Multicollinearity was assessed using variance inflation factors. Missing data was primarily handled through complete-case analysis, with multiple imputations by chained equations (m = 20) for sensitivity testing. Imputation models included all exposure, outcome, and significant covariates. Convergence diagnostics and pooled estimates confirmed that results were consistent between complete-case and imputed analyses.

### Derived variable definitions and calculation methods

Variables directly measured or recorded in the datasets included: Body Mass Index (BMI), Waist Circumference (Waist), Glucose (Glu), Glycated Hemoglobin (HbA1C), Red Blood Cell Count (RBC), White Blood Cell Count (WBC), Platelet Count (PLT), C-Reactive Protein (CRP), Total Cholesterol (TC), Triglycerides (TG), Albumin (ALB), Alkaline Phosphatase (ALP), Alanine Aminotransferase (ALT), Aspartate Aminotransferase (AST), Gamma-Glutamyl Transferase (GGT), Creatinine (CREA), Urea (UREA), Uric Acid (Urate), Lymphocyte Count (LYM), Monocyte Count (MONO), Neutrophil Count (NEU), High-Density Lipoprotein Cholesterol (HDL_C), Vitamin K (Vit_K), Retinol (Retinol), Alpha-Carotene (Alpha_carotene), Beta-Carotene (Beta_carotene), Beta-Cryptoxanthin (Beta_crytoxanthin), and Hemoglobin (HB).

Mean Corpuscular Hemoglobin (MCH) was calculated as 10× Hemoglobin (g/dL) divided by RBC (10^12^/L) and Mean Corpuscular Hemoglobin Concentration (MCHC) was calculated as 100× Hemoglobin (g/dL) divided by Hematocrit (%). The Triglyceride-Glucose Index (TyG) was defined as the natural logarithm of fasting triglycerides (mg/dL) multiplied by fasting glucose (mg/dL) divided by two, and the BMI-adjusted TyG (TyG_BMI) was obtained by multiplying TyG by BMI. The Fibrosis-4 score (FIB-4) was computed as [Age (years) × AST (U/L)]/[Platelet count (10⁹/L) × √ALT (U/L)], while the Hepatic Steatosis Index (HSI) was calculated as 8 × (ALT/AST) + BMI + 2 (if female) + 2 (if diabetes present). The Nonalcoholic Fatty Liver Disease Fibrosis Score (NFS) was estimated as −1.675 + 0.037 × Age (years) + 0.094 × BMI + 1.13 × (Impaired Fasting Glucose or Diabetes = 1, else 0) + 0.99 × (AST/ALT) − 0.013 × Platelet (10⁹/L) − 0.66 × Albumin (g/dL). The Neutrophil-to-Lymphocyte Ratio (NLR) was defined as NEU divided by LYM. The Fat-to-Energy Ratio (Fat_Energy_ratio), Protein-to-Energy Ratio (Protein_Energy_ratio), and Carbohydrate-to-Energy Ratio (Carb_Energy_ratio) were calculated as 9 × Fat (g)/Total Energy (kcal), 4 × Protein (g)/Total Energy (kcal), and 4× Carbohydrate (g)/Total Energy (kcal), respectively. The Monounsaturated Fat-to-Total Fat Ratio (MUFA_Fat_ratio) was defined as MUFA (g)/Total Fat (g). The BVI was defined as the meaning of (Vitamin B6/1.3 mg) and (Vitamin B12/2.4 μg), using 1.3 mg and 2.4 μg as reference intakes.

### Statistical analysis and robustness checks

Association analyses used cohort-appropriate models. In the UKB, Cox proportional hazards models were used to estimate the association between AVI and incident AMD. Proportional hazards assumptions were checked using Schoenfeld residuals, and time was measured in years since baseline. In NHANES and the Tianjin cohort, multivariable logistic regression was used to estimate the association between AVI and AMD status. NHANES survey weights were considered in sensitivity analyses. All models were fitted sequentially according to Model 1, Model 2, and Model 3, with continuous covariates retained in their constant form whenever possible.

Dose-response and threshold exploration proceeded through cohort-specific quartiles of AVI, with tests for linear trend, along with restricted cubic splines to evaluate nonlinearity. Knot number and placement followed conventional percentiles and were varied in sensitivity analyses.

Prespecified subgroup analyses were conducted for age, sex, hypertension, and diabetes. Interaction terms on the multiplicative scale were tested to assess effect heterogeneity. To examine variable structure and potential collinearity, we computed Spearman correlation matrices before modeling.

For prediction modeling, we applied the Boruta random forest wrapper and the least absolute shrinkage and selection operator (LASSO) for feature selection within each cohort^60^. Boruta tuning parameters were selected within predefined ranges, and LASSO was employed with 10-fold cross-validation, with a preference for the parsimonious penalty. We constructed multivariable models and derived nomograms for individualized risk estimation.

Model performance and clinical utility were evaluated using stratified training and test splits within each cohort. Discrimination was assessed by the area under the receiver operating characteristic curve. Calibration was examined with calibration plots and the Brier score. Clinical utility was quantified using decision curve analysis. Internal validation used bootstrap resampling.

Machine learning models were trained in parallel, including logistic regression, support vector machines, random forests, and gradient-boosted trees (XGBoost)^61^. Non-tree models used standardized inputs, and class imbalance was addressed with class weighting or undersampling. Performance was evaluated using the same train-test splits. Model interpretability was examined using Shapley additive explanations to quantify global and individual feature contributions and to verify the stability of age and AVI across different algorithms. Model performance was quantified using the area under the ROC curve (AUC), the Brier score, and calibration slope. Each cohort was randomly split into training (70%) and testing (30%) subsets, with bootstrap resampling (1000 iterations) used for internal validation. Decision curve analyses were performed across threshold probabilities ranging from 0.05 to 0.50, reflecting clinically meaningful ranges for early AMD screening.

Sensitivity analyses varied AVI grouping and spline knots, changed energy adjustment strategies, applied alternative missing-data procedures, and, for NHANES, incorporated survey weights and repeated analyses across alternative covariate sets.

### Ethics

The study adhered to the principles outlined in the Declaration of Helsinki. Use of UKB and NHANES followed their respective data use agreements. The Tianjin cohort obtained written informed consent at enrollment and received approval from the Tianjin Eye Hospital Ethics Committee.

### Software

Analyses were conducted in R and Python. Key R packages included survival, rms, splines, mgcv, Boruta, glmnet, pROC, and rmda. Tree-based modeling and explanations utilized XGBoost, Random Forest, and SHAP-related tools. Two-sided statistical significance was set at a p-value of 0.05.

## Supplementary information

Supplementary information

## Data Availability

The UK Biobank data used in this study are available upon approval of an application to the UK Biobank. The NHANES 2005–2008 datasets are publicly accessible from the United States Centers for Disease Control and Prevention. The Tianjin cohort data are available from the corresponding author upon reasonable request, subject to approval by the Tianjin Eye Hospital Ethics Committee. All statistical scripts and code used to generate figures and tables are available from the corresponding author upon request.
