# Supplementary material for: Antioxidant vitamin index and risk of age-related macular degeneration: multicenter validation and clinical translation
Source: NPJ Aging. 2026 Feb 21;12(1):48. doi: 10.1038/s41514-026-00348-y (PMC13040019; doi:10.1038/s41514-026-00348-y)
Supplement: Supplementary file 1 — Supplementary information [file 41514_2026_348_MOESM1_ESM.pdf]

Supplement

Table S1. Dietary variables, units, and data sources

Dietary variables, units, and data sources				
**Variable**	**Unit (per day)**	**From food (24- h recall)***	**From supplements (30- day inventory)****	**Total intake (analysis)**
Energy	kcal	Mean of two non- consecutive 24- h recalls (AMPM/ WebQ)	Not collected	Food mean
Protein	g	Mean of two non- consecutive 24- h recalls	Not collected	Food mean
Carbohydrate	g	Mean of two non- consecutive 24- h recalls	Not collected	Food mean
Total fat	g	Mean of two non- consecutive 24- h recalls	Not collected	Food mean
Total MUFA (monounsaturated fat)	g	Mean of two non- consecutive 24- h recalls	Not collected	Food mean
Vitamin A	µg RAE	Mean of two non- consecutive 24- h recalls	Daily average: per- unit content × units per intake × frequency/day; units harmonized to µg RAE	Food mean + supplement daily
Vitamin B6	mg	Mean of two non- consecutive 24- h recalls	Daily average as above (mg)	Food mean + supplement daily
Vitamin B12	µg	Mean of two non- consecutive 24- h recalls	Daily average as above (µg)	Food mean + supplement daily
Vitamin C	mg	Mean of two non- consecutive 24- h recalls	Daily average as above (mg)	Food mean + supplement daily
Vitamin E (α- tocopherol equiv.)	mg α- TE	Mean of two non- consecutive 24- h recalls	Daily average as above; units harmonized to mg α- TE	Food mean + supplement daily
Vitamin K (primarily phylloquinone)	µg	Mean of two non- consecutive 24- h recalls	Daily average as above (µg)	Food mean + supplement daily

Table S2 24-Hour Dietary Recall Record Form (Day 1 / Day 2)

24-Hour Dietary Recall Record Form (Day 1 / Day 2)

Participant ID: ___________________________

Date of recall: ____ / ____ / ________ **Day of week:** ☐ Mon ☐ Tue ☐ Wed ☐ Thu ☐ Fri ☐ Sat ☐ Sun

Type of interview: ☐ In-person ☐ Telephone ☐ Online (WebQ)

Interviewer ID: _____________ **Participant initials:** _____________

Did you take any supplements or vitamins today? ☐ Yes ☐ No

If yes, list below in the supplement section.

Section A. Meal-by-Meal Record

Time (HH:MM)	Meal / Occasion<br>(Breakfast, Lunch, Dinner, Snack, Beverage, etc.)	Location / Setting<br>(Home, Work, Restaurant, School, Street, etc.)	Food / Beverage Name<br>(Include brand, preparation, main ingredients)	Cooking Method<br>(Boiled, Steamed, Stir-fried, Deep-fried, Roasted, etc.)	Portion Size / Quantity<br>(g, mL, cup, slice, piece, bowl, spoon, etc.)	Accompanying Foods / Sauces / Condiments	Fat / Oil Type & Amount Used	Added Sugar / Salt / Seasoning	Remarks<br>(Leftovers, special recipe, fasted, etc.)

Section B. Between-Meal Items (Snacks, Drinks, Supplements)

Time	Item Type (Snack / Beverage / Vitamin / Supplement)	Brand / Product Name	Serving Size	Number of Servings	Frequency per Day / Week	Preparation / Ingredients	Remarks

Section C. Daily Summary

1. Did you eat or drink anything before going to bed (after 9 p.m.)? ☐ Yes ☐ No If yes, specify: ____________________________

2. Did you skip any usual meal? ☐ Yes ☐ No If yes, which one? ____________________________

3. Were there any unusual events (travel, illness, celebration) on this day? ☐ Yes ☐ No

4. Appetite level: ☐ Normal ☐ Less than usual ☐ More than usual

5. Any food allergies or intolerances? ☐ Yes ☐ No If yes, list: _______________________________

6. Cooking at home: ☐ Self prepared ☐ Family member ☐ Purchased (restaurant/ready-to-eat)

Section D. Supplement and Vitamin Intake (if any)

Brand / Product Name	Formula Type (AREDS / AREDS2 / Multivitamin / Others)	Per-unit content (please copy from label)	Unit	Units per intake	Frequency<br>(per day / week / month)	Start Date	End Date	Remarks
		Vitamin A _____ (☐ μg RAE ☐ IU); source: ☐ Retinol ☐ β-Carotene<br>Vitamin E _____ (☐ mg α-TE ☐ IU); form: ☐ Natural d-α ☐ Synthetic dl-α<br>Vitamin B6 _____ mg; Vitamin B12 _____ μg; Vitamin C _____ mg; Vitamin K _____ μg						

Section E. Portion Size Reference (for participant guidance)

Common Food	Typical Household Measure	Approx. Weight (g)
Cooked rice (1 medium bowl)	1 rice bowl	200–250 g
Bread slice	1 piece	25–30 g
Egg (boiled)	1 piece	50 g
Apple (medium)	1 fruit	150–180 g
Milk / Beverage	1 cup	240 mL
Cooking oil (1 tablespoon)	15 mL	13–15 g
Butter / Margarine	1 teaspoon	5 g
Sugar	1 teaspoon	4 g

Meat (pork/beef/chicken)	1 palm-size portion	~100 g
Fish fillet	1 hand palm (no fingers)	~120 g
Nuts	1 handful	~30 g

Instructions for Interviewer / Researcher

Use AMPM five-step method: (1) quick list → (2) forgotten foods → (3) time/occasion → (4) detailed description → (5) final probe.

Prompt for: added ingredients (oil, sugar, condiments), beverages, sauces, snacks, supplements, cooking fats, portion size.

Check completeness: confirm no meals/snacks missing between waking and bedtime.

Record in grams or milliliters when possible.

For supplements, photograph or copy label information.

If uncertain portions, use household measures (spoon, handful) and add notes.

Table S3. Summary of machine-learning algorithms, hyperparameters, data split, and performance across cohorts

Algorithm	Key hyperparameters (tuning strategy)	Train:test split	Test AUC – UKB	Test AUC – NHANES	Test AUC – Tianjin
Logistic regression	Linear model with L2 regularization; class_weight = "balanced"; regularization strength C selected by cross- validated grid search (e.g., C ∈ {0.01, 0.1, 1, 10}).	70:30	0.765	0.755	0.833
Support vector machine (SVM)	Radial basis function (RBF) kernel; probability estimates enabled; C and γ tuned by cross- validated grid search over logarithmic ranges.	70:30	0.635	0.683	0.857
Random forest	Ensemble of decision trees; number of trees tuned in a predefined range (e.g., 200–1,000); maximum depth, minimum samples per split/leaf chosen by cross- validation; class_weight = "balanced".	70:30	0.784	0.763	0.842
Extreme gradient boosting (XGBoost)	Gradient- boosted decision trees; learning rate, number of estimators, maximum depth, subsampling rate, and column sampling rate tuned by cross- validated grid search; early stopping based on validation loss.	70:30	0.787	0.783	0.848

Figure S1

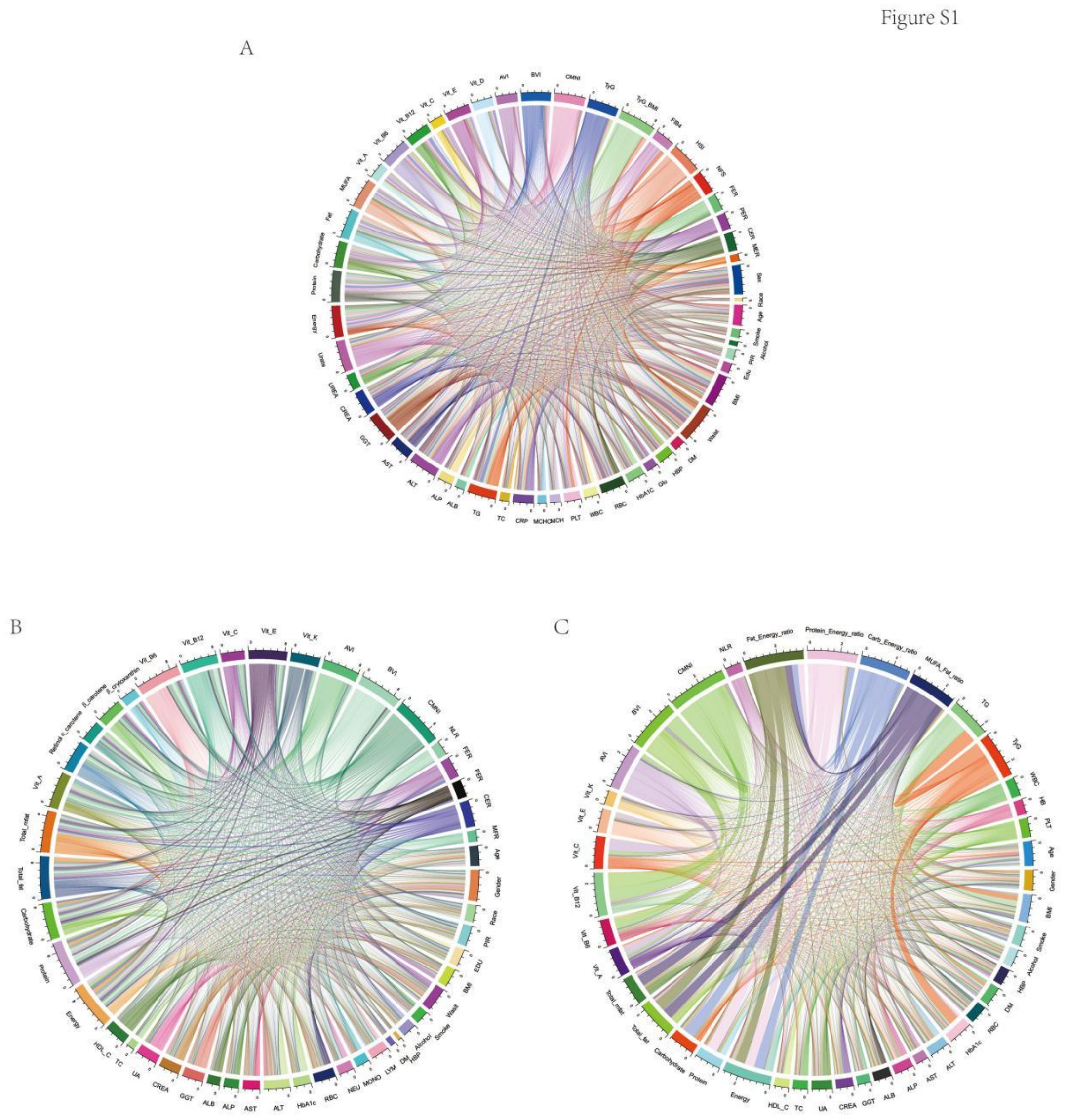

Figure S1. Spearman correlation networks across cohorts.

Chord diagrams show the relationships among nutritional and biochemical variables in (A) UKB, (B) NHANES, and (C) Tianjin cohorts. AVI is strongly and positively correlated with Vitamins A, C, and E and moderately with macronutrient intake, indicating coherent biological structure across populations.

Figure S2

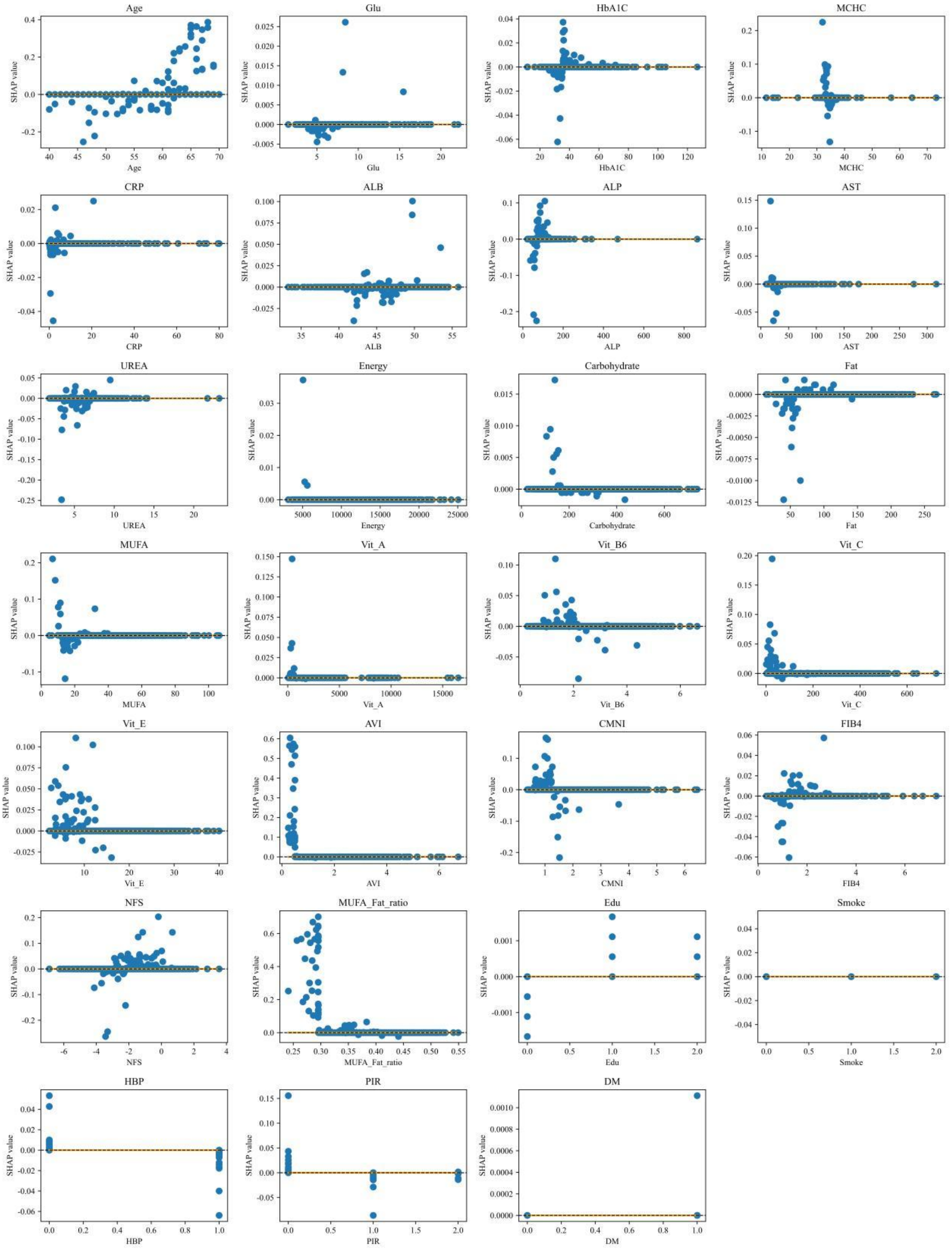

Figure S2. SHAP value distribution plots in UKB.

Individual SHAP values show the contribution of each variable to AMD prediction in the UKB model.

Figure S3

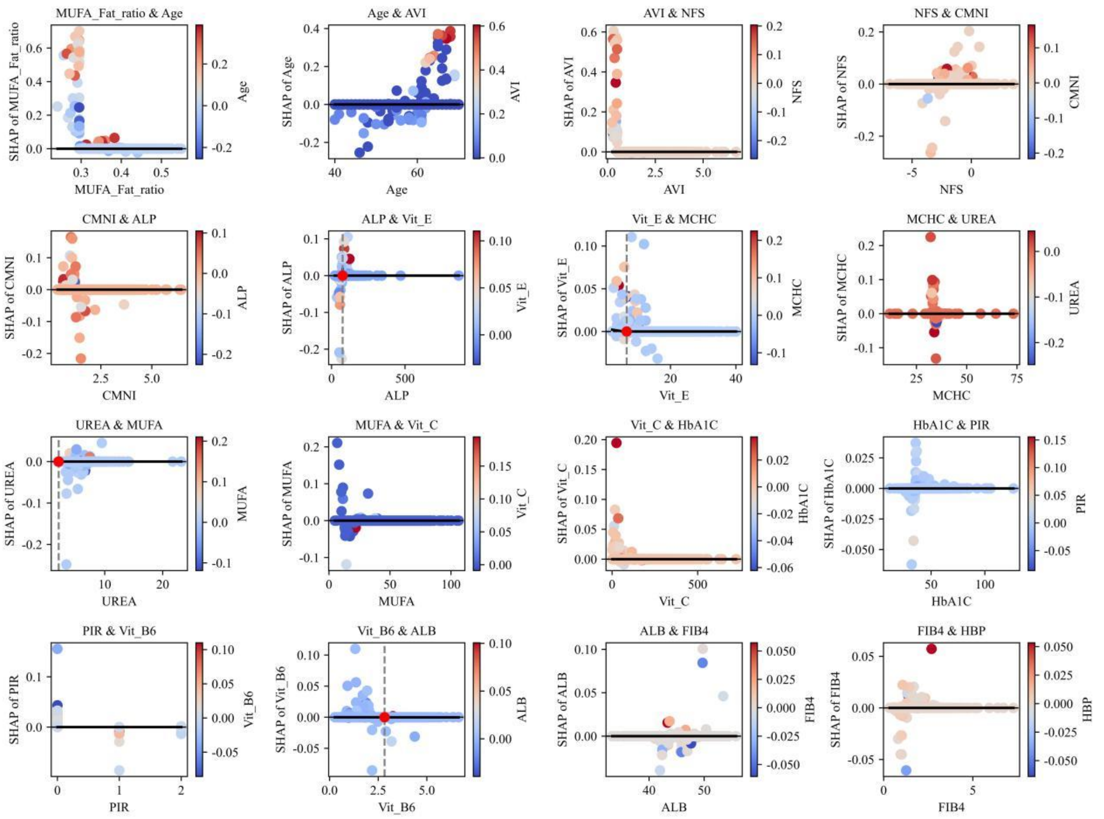

Figure S3. SHAP interaction plots in UKB.

Pairwise SHAP dependence analyses illustrate interactions between key predictors such as AVI, MUFA ratio, NFS, and other nutritional factors.

Figure S4

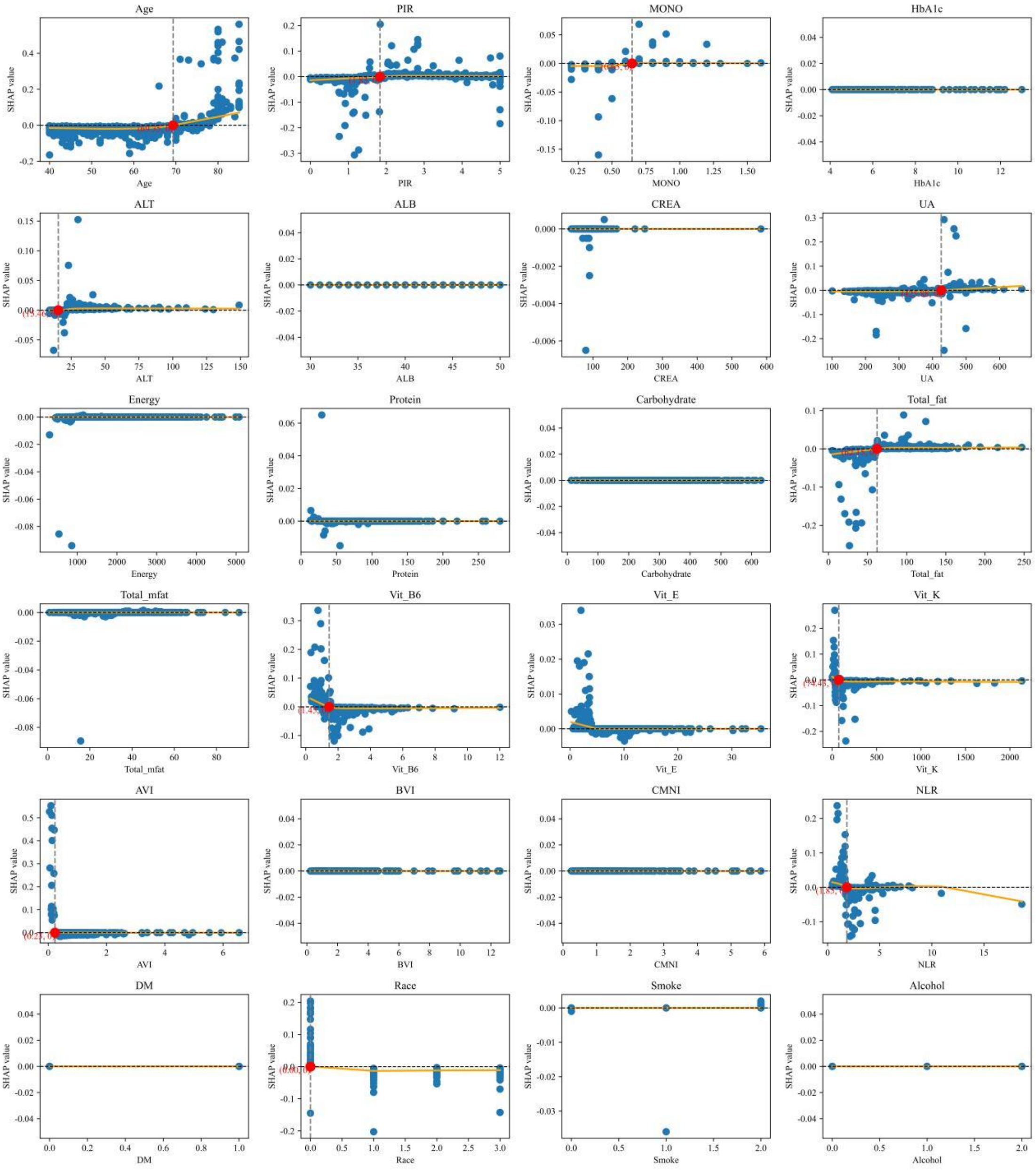

Figure S4. SHAP value distribution plots in NHANES.

SHAP results from NHANES demonstrate variable effects and consistency of AVI and age as main predictors.

Figure S5

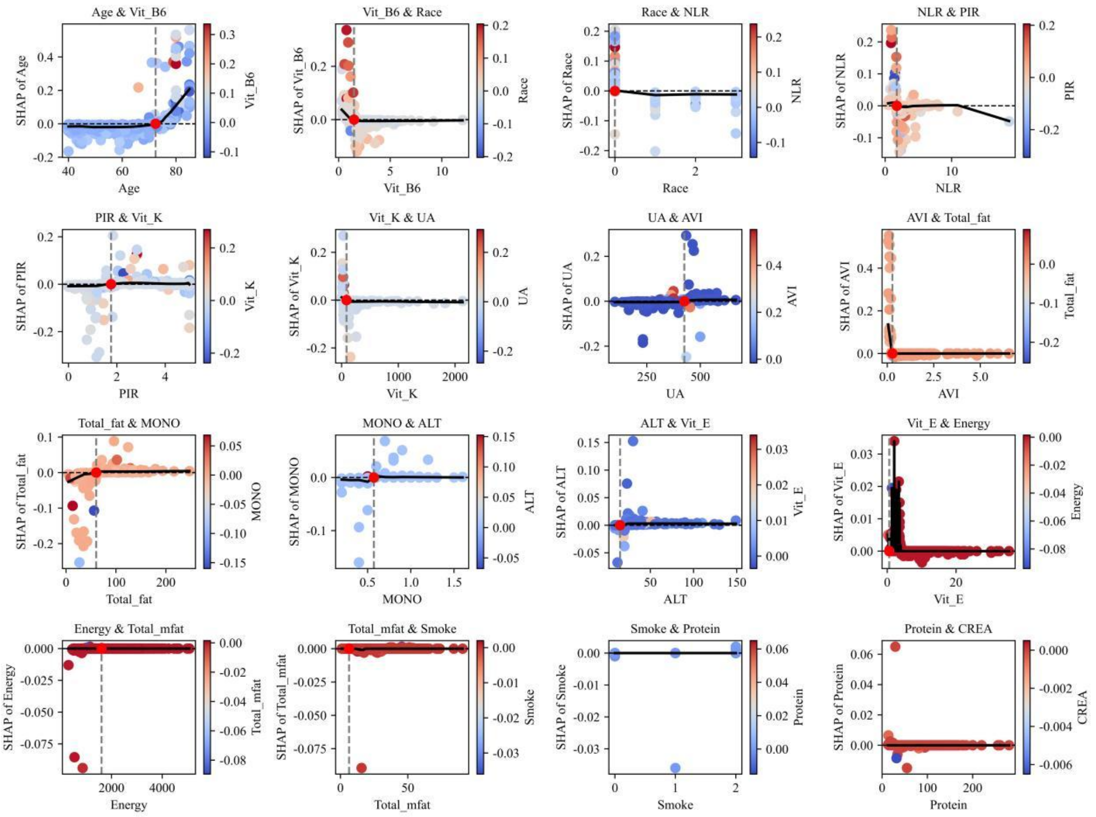

Figure S5. SHAP interaction plots in NHANES.

Two-dimensional SHAP interactions highlight the combined effects of antioxidant and metabolic indicators on AMD risk.

Figure S6

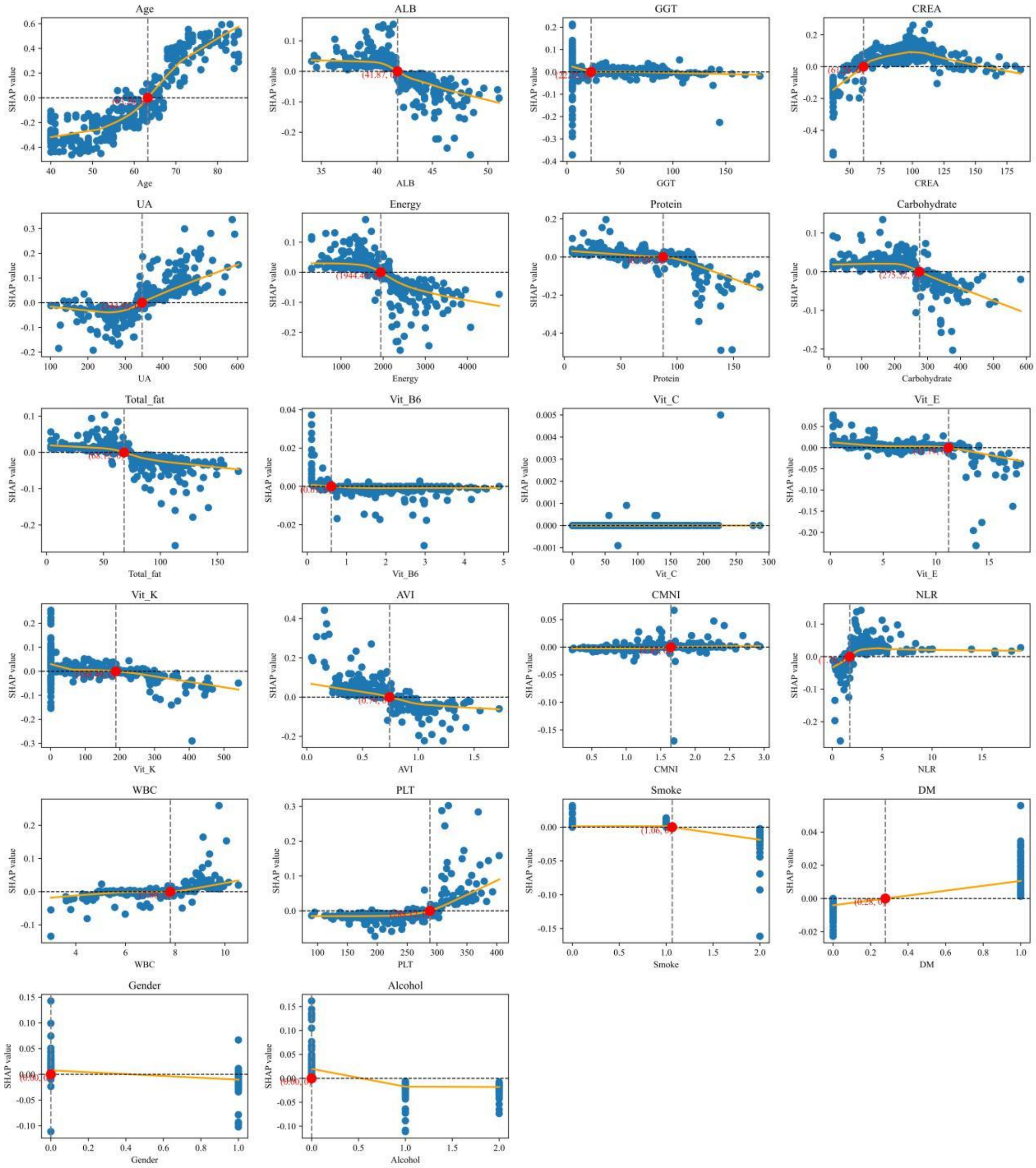

Figure S6. SHAP value distribution plots in the Tianjin cohort.

Feature contributions derived from the Tianjin model show age and AVI as dominant determinants with supporting metabolic factors.

Figure S7

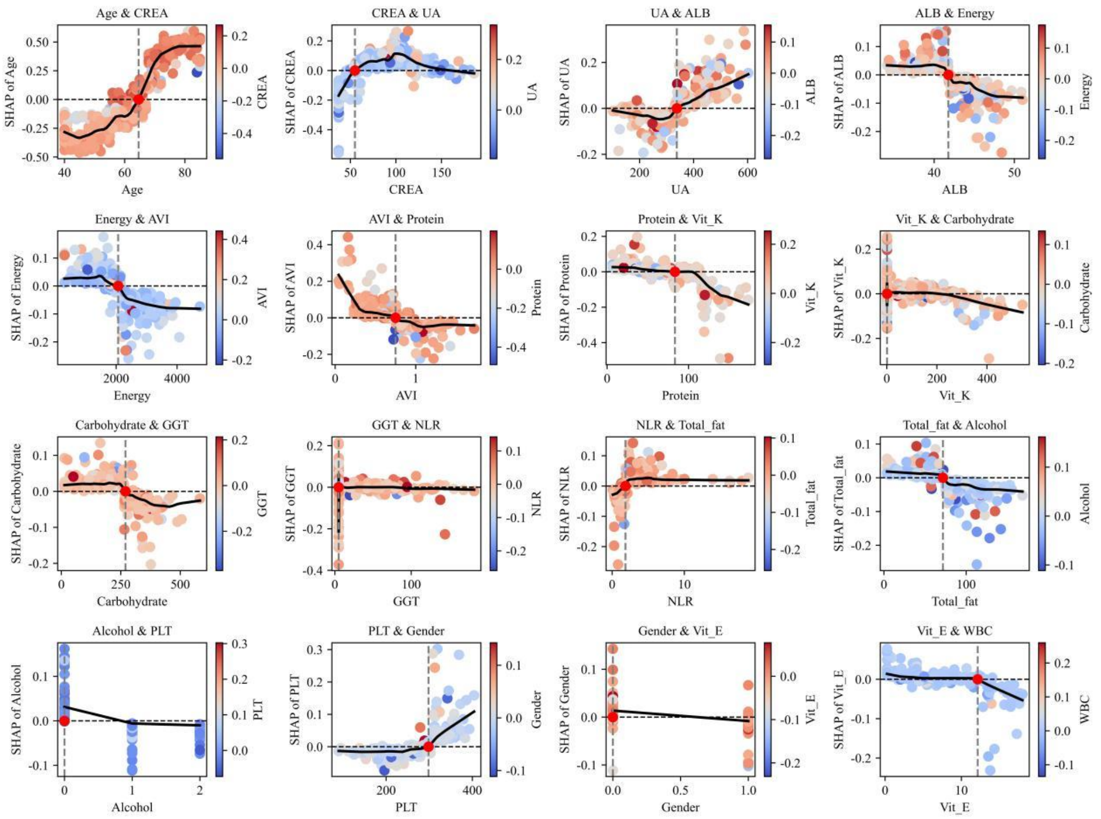

Figure S7. SHAP interaction plots in the Tianjin cohort.

Variable interaction analyses reveal synergistic effects between AVI and nutritional, metabolic, and inflammatory markers in AMD prediction.

Figure S8

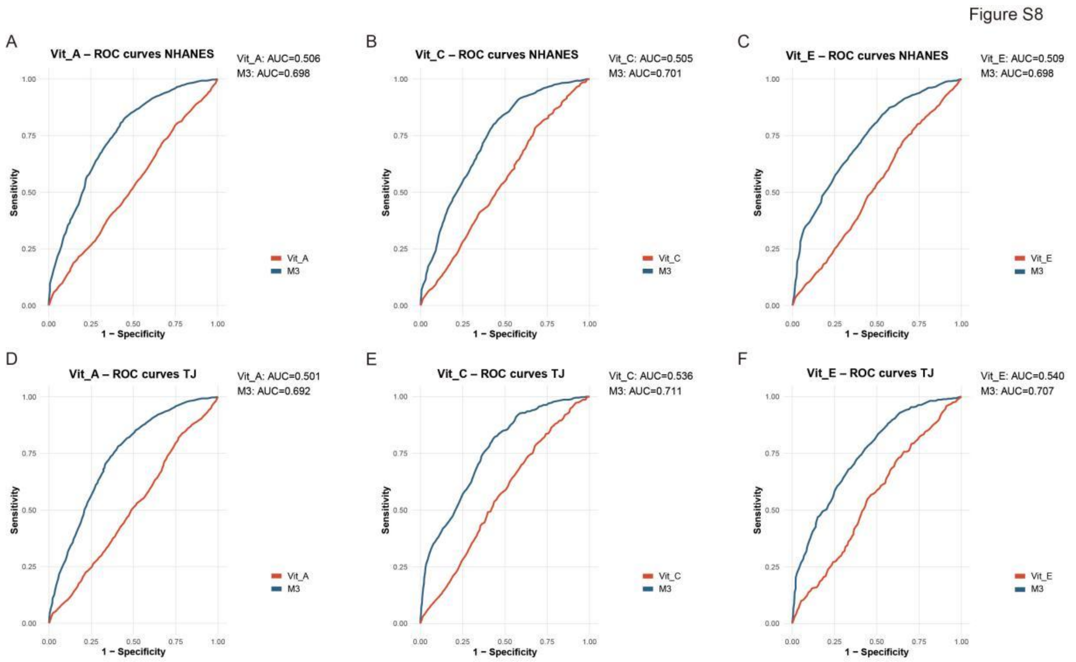

Figure S8. ROC analysis of single antioxidant vitamins and Model 3 without AVI.

Receiver operating characteristic (ROC) curves for vitamin A, vitamin C, and vitamin E modeled individually as the sole antioxidant exposure within the fully adjusted Model 3 framework in the NHANES cohort (A–C) and the Tianjin cohort (D–F). In each panel, the red curve represents the single-vitamin model, while the blue curve represents the corresponding Model 3 including that vitamin alone, without the Antioxidant Vitamin Index (AVI) or the other two vitamins.
